# In Situ Synthesis of Non-Cytotoxic Tellurium Nanoparticle and Methacrylate Photopolymer Resin Composite with Antibacterial Activity

**DOI:** 10.3390/polym17202735

**Published:** 2025-10-12

**Authors:** Dmitriy A. Serov, Aleksandr V. Simakin, Dmitriy E. Burmistrov, Ilya V. Baimler, Pavel P. Chapala, Maxim E. Astashev, Fatikh M. Yanbaev, Valeriy A. Kozlov, Sergey V. Gudkov

**Affiliations:** 1 Prokhorov General Physics Institute of the Russian Academy of Sciences, Vavilov Str. 38, 119991 Moscow, Russia; dmitriy_serov_91@mail.ru (D.A.S.); avsimakin@gmail.com (A.V.S.); dmitriiburmistroff@gmail.com (D.E.B.); ilyabaymler@yandex.ru (I.V.B.); astashev@yandex.ru (M.E.A.); v.kozlov@hotmail.com (V.A.K.); 2 HARZ Labs LLC, Silikatnaya Str. 51A, bld.5, 141013 Mytischi, Russia; p.chapala@harzlabs.com; 3 HARZ Labs DOO, Aleksandra Aca Prijića 16A, 81101 Podgorica, Montenegro; 4 Federal Research Center “Pushchino Scientific Center for Biological Research of the Russian Academy of Sciences”, Institute of Cell Biophysics of the Russian Academy of Sciences, 3 Institutskaya St., 142290 Pushchino, Russia; 5 Federal Research Center Kazan Scientific Center of the Russian Academy of Sciences, ul. Lobachevskogo 2/31, Tatarstan, 420088 Kazan, Russia; f.yanbayev@knc.ru; 6 Department of Fundamental Sciences, Bauman Moscow State Technical University, 5 2nd Baumanskaya St., 105005 Moscow, Russia

**Keywords:** tellurium nanoparticles, poly(methyl methacrylate), mechanical properties, Jacobs working curves, ROS, 8-oxoguanin, long-lived reactive protein species, antibacterial, cytotoxicity

## Abstract

Methacrylate photopolymer resin (MPR) is widely used in various fields, including the biomedical field. There are several problems associated with their use: the potential toxicity of monomer residues during incomplete polymerization and the possibility of bacterial expansion. Doping polymers with nanoparticles is one of the ways to increase the degree of polymerization (protection from toxicity), improve the performance characteristics of the polymer, and add antibacterial properties. We used an in situ polymerization method to obtain the composites of MPR with tellurium nanoparticles (TeNPs) with a dopant concentration of 0.001, 0.01, or 0.1% (*v*/*v*). The composite of MPR+TeNPs had a higher degree of polymerization compared to MPR without NPs, improved mechanical properties, and pronounced antibacterial activity. The effects depended on the concentration of TeNPs. All of the studied composites had no cytotoxic effect on human cells. MPR+TeNPs 0.1% had the maximum antibacterial effect, which is probably realized through Te-dependent induction of oxidative stress (increase in the generation of 9-oxoguanine and long-lived reactive forms of proteins). The results obtained deepen the knowledge about the influence of NPs of leading metals on photopolymerization and the final properties of the methacrylate matrix, and the synthesized MPR+TeNP composites may find potential biomedical applications in the future.

## 1. Introduction

Additive technologies and 3D printing make it possible to produce complex shapes and surfaces that are very difficult to obtain using conventional methods [1]. Currently, 3D printing is already used in more than a dozen areas of human economic activity: the production of stainless steel frames, fine robotics, the creation of bone tissue scaffolds, artificial heart valves, the development of drug delivery systems, and others [2,3,4]. The global 3D printing market size in 2023 was >USD 19.8 billion, with a projected CAGR of ~21.2%, indicating the high relevance of research in this area [5]. Despite the high growth rates of the industry’s volumes and applications, there are a large number of problems and challenges that are especially acute in biomedical applications. The share of printing using polymer materials is about 20% of the total market, which confirms the need to find new, efficient, and inexpensive solutions for polymer 3D printing.

Bath photopolymerization (BPP) occupies a special place in additive manufacturing due to its high accuracy and printing speed, a wide range of source materials, and relatively low production costs [6,7,8]. 3D printing and the use of composite materials are finding increasing applications in regenerative biomedicine and the development of antibacterial materials [9,10]. Methacrylate photopolymer resin (MPR) is similar to polymethyl methacrylate. Products made from MPR have already been approved for use in various areas of modern medicine, including surgery, prosthetics, dentistry, orthopedics, and other areas [11,12,13,14,15]. Objects created for medical use must be non-toxic to humans, have the required mechanical properties throughout the entire service life of the object [16,17,18]. Photopolymer resins consist of a set of precursor monomers and a photoinitiator. As a rule, it is impossible to completely use up the original components during photopolymerization, and their residues can have a toxic effect. The problem of removing “unused” monomers and/or increasing the degree of polymerization arises [19,20]. The introduction of metal and non-metal NPs into the photopolymer resin can significantly increase the rate and efficiency of BPP. Among the mechanisms for accelerating BPP are increased absorption and scattering of light, photocatalysis, electron transfer, surface interaction, and light [21,22,23,24,25,26]. Thus, the introduction of NPs can help ensure a high degree of polymerization of photopolymer resins and the biosafety of the resulting products.

Unfortunately, biosafe and biocompatible materials can be a favorable substrate not only for eukaryotic cells, but also for colonization by bacteria, and can become a potential source of infections [27,28,29,30]. The problem of bacterial infections is complicated by the development of bacterial resistance to antibiotics, so it is necessary to search for effective, inexpensive, and long-acting alternative solutions [31]. Adding metal and non-metal NPs to a polymeric material is one way to impart antibacterial properties to them without the use of antibiotics [32,33,34,35,36,37]. The mechanisms of antibacterial activity are common to a wide range of metal and non-metal NPs and include the generation of reactive oxygen species (ROS), direct or indirect genotoxicity, inhibition of bacterial enzymes leading to a violation of metabolic processes, disruption of the integrity of the cell wall, and a number of others [38,39,40,41,42,43,44].

In addition, the introduction of NPs of metals, non-metals, and semi-metals can change the following properties of the polymer matrix MPR: temperature relaxation, high temperature resistance, maximum tensile strength, with the corresponding strain, Shore D hardness, refractive index, light scattering and absorption, electrical conductivity, etc. [45,46,47,48,49,50,51].

Tellurium semi-metal nanoparticles (TeNPs) have a variety of applications due to their unique properties. They are used in electronics, optoelectronics, biomedicine, and environmental protection, for example, in field-effect transistors, photodetectors, sensors, antibacterial agents, drug delivery systems, etc. [52,53,54,55]. Modification of polymers with tellurium or TeNPs is a young but rapidly developing area of materials science, including in biomedical applications. Changes in the redox, optical, electrical, mechanical, and other properties of polymers after the inclusion of Te in the polymer matrix have been described [56,57,58]. In addition, the introduction of TeNPs increases the antibacterial properties of polymer materials [59,60,61,62]. NPs can be introduced in MPR matrices via several ways: simultaneous polymerization of MPR and NP co-precipitation, addition of NPs into a heated polymer (above the glass transition temperature), and in situ polymerization of a mixture of NPs and precursors [63,64,65,66]. We chose the in situ polymerization method since it does not require heating the polymer, removing solvents, and, as mentioned above, should provide an increase in the degree of polymerization. As a method for synthesizing TeNPs, we chose the method of laser ablation in water because this method does not require additional chemical reagents and allows us to obtain NPs with narrowly distributed sizes and a uniform shape [67].

The aim of this study was to evaluate the potential of TeNPs added at different concentrations in improving the mechanical properties and degree of polymerization of MPR, as well as to evaluate the generation of reactive oxygen species and markers of biopolymer damage (8-oxoguanin and long-lived protein species—LRPSs) in the presence of MPR+TeNP composites, and the antibacterial and cytotoxic properties of MPR+TeNPs.

## 2. Materials and Methods

### 2.1. Synthesis of TeNPs

**Figure 1:**
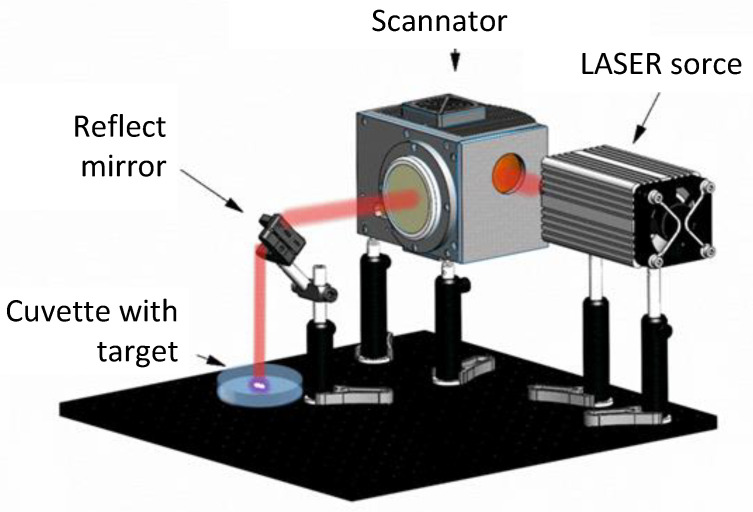
Schematic representation of the setup for obtaining NPs by laser ablation in solution.

TeNPs were synthesized by laser ablation of a solid Te target (99.97%) in water [68]. The general scheme of the setup is presented below (Figure 1). The Nd:YaG laser source model NL 210 SH (Ekspla, Vilnius, Lithuania) was used. The following parameters were used for ablation: λ_1_ = 1064 nm, λ_2_ = 532 nm, *f* = 1 kHz, τ = 4 ns, and E_p_ = 1.5 mJ. The laser beam diameter at the waist was 100 μm. Deionized water with an electrical conductivity not exceeding 0.1 μS/cm was used as the working fluid; the volume of the working fluid was 100 mL. The target irradiation time was 30 min. During irradiation, the radiation beam moved along the target surface using a galvanomechanical scanner (LScanH, Ateco-TM, Moscow, Russia) and an F-theta lens. The beam trajectory was several parallel lines inscribed in a square. The line filling resolution was 70 lines/mm. The radiation beam movement speed was 3000 mm/s. The synthesized nanoparticles were centrifuged with CPS DC24000 disc centrifuge (CPS Instruments, Prairieville, LA, USA) and collected from the selected area so that their size distribution was as uniform as possible.

### 2.2. Physicochemical Characteristics of Received Nanoparticles

Distribution of NPs by size and ζ-potential were evaluated by dynamic light scattering (DLS) with Malvern zetasizer ultra analyzer in multi-angle dynamic light scattering more (Malvern Panalytical Ltd., Malvern, UK) in quartz cuvettes with ‘Dip’ Cell ZEN1002 electrode (Malvern Panalytical Ltd., Malvern, UK). ZS Xplorer software v. 3.2 (Malvern Panalytical Ltd., Malvern, UK) was used for primary data processing. UV-visible absorption spectra of water TeNP colloids was registered with two-beam CINTRA 4040 spectrometer (GBC Scientific Equipment Pty Ltd., Keysborough, VIC, Australia). The optical density was measured in the range of 200–800 nm with a step of 1 nm. The spectra were measured in quartz cuvettes with a volume 3 mL. The optical path length was 1 cm. The absorption spectra of the working liquid used in laser ablation were used as reference spectra. To obtain TEM images of particles and study their morphology, a Libra 200 FE HR transmission electron microscope (Carl Zeiss, Jena, Germany) was used. Gold microscopy grids were used to prepare nanoparticles for TEM microscopy.

### 2.3. Preparation of Nanocomposite MPR+TeNP Material

**Figure 2:**
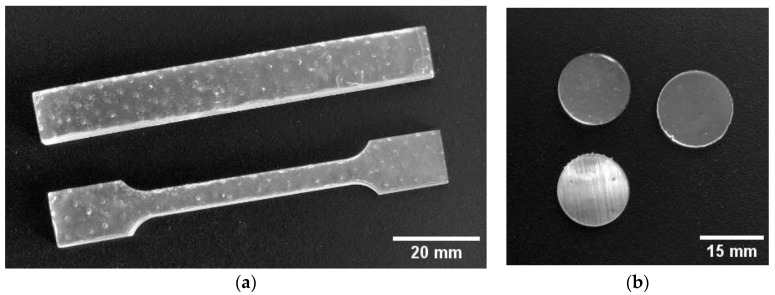
Photos of MPR+TeNPs 0.1% samples for mechanical (**a**) and cytotoxic (**b**) assays.

The method for integrating TeNPs into MPR consisted of two main steps: preparing NP dispersions in acetone and introducing the NPs into the polymer matrix. The solvent was replaced by centrifuging 40 mL of the TeNP suspension three times in a 3-16KL centrifuge (Sigma-Aldrich, St. Louis, MO, USA) for 40 min at 7000 g and then replacing the entire solvent volume with 99.5% acetone (Khimmed, Moscow, Russia). Between centrifugations, the particles were dispersed in the added solvent for 3–5 min of treatment in Ultrasonic bath PS-20A (Digital Pro, Haidian District, Beijing, China) at 40 kHz, with 3–5 min of stirring. Then, TeNPs in acetone were added to the MPR at a mass fraction of 0.001, 0.01, or 0.1% (*v*/*v*). Photopolymer Clear Pro resin (Harz Labs, Mytishchi, Russia) was used as polymer matrices. This PMR corresponds to safety standard ISO 10993-18 [69] and is approved as material for medical purposes in the Russian Federation (registration certificate for a medical device No. RZN 2020/12007). For printing of test samples from control MPR and MPR+TeNPs, 3 Ultra 12K MSLA Saturn (Elegoo, Shenzhen, China) printer was used. For each of the resulting composites and the NP-free MPR, ISO 179-1:2023 [70] Type 1 and ISO 527-2:2025 Type 1A [71] test specimens were printed to test the mechanical properties (Figure 2). For cytotoxic and microbiological studies, round plates with a thickness of 0.5 mm and Ø 16 and 8 mm, respectively, were printed. To assess the generation of ROS in water, plates measuring 10 × 10 × 0.5 mm were made.

After the photopolymer printing cycle, the samples were separated from the platform and washed in absolute isopropyl alcohol 99.9% (Lenreaktiv, Moscow, Russia) for 6 min using a wash bath mounted on the magnetic platform of the UW-02 3D cleaning and curing device (Creality3D, Shenzhen, China) [72,73].

### 2.4. Study of the Composition and Microrelief of the Surface

The microrelief of the obtained composites was studied by atomic force microscopy (AFM) in non-contact and semi-contact modes with NT-MDT microscope (LLC, Zelenograd, Russia) without preliminary sample preparation. The refractive index of polymethacrylate is ~1.5, and Te is ~2.3. The large difference in refractive indices allows the use of modulation interference microscopy (MIM) to assess the distribution of NPs in the thickness of the polymer matrix. This study was performed using an MIM-321 microscope with an operating wavelength of 632 nm (Amphora Labs, Moscow, Russia). The effect of doping MPR by TeNPs was evaluated for the chemical composition of the matrix using UV-vis and FTIR spectra. FTIR spectra of materials were recorded with IR-8000 FTIR spectrometer (SAS LLC, Krasnoyarsk, Russia) equipped by a ZnSe Sealed Flat Plate (Pike Technologies, WI, USA). UV-vis spectra were recorded at 200–800 nm with two-beam spectrometer Cintra 4040 (GBC Scientific Equipment Pty Ltd., Victoria, Australia).

### 2.5. Study of Strength Characteristics

Tensile and bending tests of printed specimens Type 1A and Type B (ISO 527-2:2025) [71] were evaluated using a WDW-5S universal testing machine (Hongtuo, Binzhou, China). The bending tests were conducted according to ASTM D790, and the tensile tests were conducted according to ASTM D638-22 standard [74].

### 2.6. Evaluation of ROS Generation in Aqueous Solutions in the Presence of MPR+TeNPs

The influence of MPR+TeNP composites or MPR without NPs on the rate of generation of hydrogen peroxide (H_2_O_2_) and hydroxyl radicals (^•^OH) in water was assessed using chemiluminescence and fluorimetric methods, as described in detail earlier [75,76]. To measure the H_2_O_2_ concentration, samples (10 × 10 × 0.5 mm) were placed in distilled water and incubated for 3 h at 40 °C. After incubation, 50 μ M para-iodophenol, 50 μM luminol, and 10 nM horseradish peroxidase (in 1 mM Tris-HCl buffer, pH 8.5) were added. The H_2_O_2_ concentrations were estimated by the intensity of chemiluminescence using a highly sensitive chemiluminometer “Biotox-7A-USE” (Engineering Center-Ecology, Russia). The lower limit of detection of H_2_O_2_ was <0.1 nM. To determine the OH- radicals concentration, the samples were placed in an aqueous solution of CCA in a phosphate buffer and incubated at a temperature of 80.0 ± 0.1 °C for 2 h. The fluorescence intensity of 7-hydroxycoumarin-3-carboxylic acid (product formed during the reaction) was recorded using a spectrofluorimeter JASCO 8300 at excitation and emission wavelengths of 400 and 450 nm, respectively. In all experiments, control measurements were performed without samples to exclude background signals. Each analysis was performed in triplicate.

### 2.7. Quantitative Analysis of Macromolecular Damage (Generation of 8-Oxoguanine and Long-Lived Reactive Proteins Species)

Determination of 8-oxoguanine concentrations in DNA samples was performed by ELISA with specific primary monoclonal anti-8-oxoguanine antibodies (diluted 1:2000). A solution of 350 μg/mL DNA was denatured by heating in a water bath for 5 min and then cooling on ice. Volumes of 42 μL of this solution were taken for analysis. DNA immobilization was performed for 3 h at 80 °C. Non-specific binding sites were blocked by a 1% solution of non-fat dry milk in Tris-HCl buffer (pH 8.7) with the addition of 0.15 M NaCl for 14–18 h at room temperature. Incubation with primary antibodies was performed for 3 h at 37 °C. After washing, secondary antibodies conjugated with horseradish peroxidase (diluted 1:1000) were added. The mixtures were incubated for 1.5 h at 37 °C. The enzymatic reaction was detected using 18.2 mM ABTS in the presence of 2.6 mM H_2_O_2_ in 75 mM citrate buffer (pH 4.2). The reaction was stopped by adding 100 μL of 1.5 mM sodium azide when color appeared [77]. Optical density was measured at 405 nm on a Feyond-A400 plate reader (Ausheng, China). To assess LRPSs generation, 10 × 10 × 0.5 mm composite material plates were incubated for 120 min in 10 mL of 0.1% aqueous BSA colloid at 40 °C. After incubation, the samples were kept in the dark at room temperature and intensity of water chemiluminescence was measured after 60, 180, and 300 min. Measurements were performed using a highly sensitive Biotox-7A chemiluminometer (Ecology Engineering Center, Moscow, Russia) in 20 mL polypropylene vials (Beckman, Brea, CA, USA) in complete darkness at 25 °C. BSA without heating was used [78].

### 2.8. Study of Antibacterial Activity

Samples (circles Ø 10 mm) of MPR or MPR+TeNPs were placed in the wells of a sterile 24-well culture plate with NP concentrations of 0.001, 0.01, or 0.1% (*v*/*v*). Each well was filled with 1 mL of *Escherichia coli* bacterial cell suspension (10^6^ cells/mL) in LB broth (DiEm, Moscow, Russia). Only bacterial suspension without samples was added to the control wells. Sterile LB broth was added to some wells to control contamination. The plate was covered with a lid and placed in a plate reader–incubator Feyond-A400 (Allsheng, Hangzhou, China) with a thermostat. To record growth curves, OD_600_ was recorded every hour for 24 h. Incubation was performed at 37 °C and with constant shaking at 200 rpm. For each experimental variant, 6 independent measurements were performed. For a more precise count of bacterial cells and assessment of the bactericidal effect of the resulting composites, the bacterial suspension was resuspended after recording the growth curves, the samples were removed, and the cells were stained with 4 μM PI (Lumiprobe, Hunt Valley, MD, USA) for 1 h in the dark at 25 °C. After staining, the cells were analyzed using a LongCyte CLQC-281 (ChBio, Nantou, Taiwan) flow cytometer. The concentration of bacterial cells in a fixed volume was calculated with the FS-SS recording mode. Count of dead bacterial cells was evaluated with the fluorescence recording mode at excitation wavelengths of 488 nm and emission wavelengths of 577 nm. Three independent measurements were performed for each experimental variant. At least 50,000 events were analyzed in each measurement. To assess the concentration of bacteria in the suspension, an automated recalculation of the number of events per 1 mL of suspension was performed. The bactericidal effect was assessed by the proportion of dead cells. The proportion of dead cells was estimated by the threshold method when setting the gate boundaries according to the geometric mean of the PI fluorescence intensity in control samples without polymers or composite materials.

### 2.9. Evaluation of Cytotoxic Effect

The cytotoxicity of polymeric material samples was tested against the human spleen fibroblast culture (HSF; the cells were kindly provided by the Collection of Human Cell Cultures for Biomedical Purposes of the VILAR). The cells were cultured and subcultured according to standard protocols in freshly prepared DMEM/F12 culture medium supplemented with 10% FBS, 2 mM L-glutamine, 25 units/mL penicillin and 25 μg/mL streptomycin (all PanEco, Moscow, Russia). On the day of the experiment, a cell suspension of 10^5^/200 μL/glass was applied to the surfaces of round cover glasses (Ø 25 mm, MiniMed, Moscow, Russia), pre-sterilized at 180 °C for 2 h, and placed on the bottom of the wells of a 6-well culture plate. Drops of cell suspension on glasses were incubated for 30 min in a CO_2_ incubator (at 37 °C, 5% CO_2_) for adhesion to the glass surface. Then, 1800 μL of warm ready-made culture medium were added to each well and one sample of the tested polymer material was immersed in culture medium. The cells were then cultured for 72 h. Cell viability, cell surface area, and nuclei area were assessed using fluorescence microscopy. The cells were stained for 30 min with 5 μM Hoechst 34580 and 2 mM propidium iodide (PI) immediately before measurement to assess viability. For cytoplasmic contrasting, 5 μM of Rhodamine-123 was used, added together with Hoechst 34580. Samples were analyzed using a DMI4000B confocal fluorescence microscope (Leica, Wetzlar, Germany) equipped with an SDU-285 digital camera (SpetsTeleTekhnika, Moscow, Russia). Fluorescence micrographs were recorded with WinFluorXE software v 3.8.7 8-12-16 (J. Dempster, Strathclyde Electrophysiology Software, University of Strathclyde) at excitation/emission wavelengths of 350/460 nm for Hoechst 34580, 488/520 nm for Rhodamine-123, and 540/590 nm for PI. The data were obtained as 12-bit monochrome images. Subsequent analysis was performed using ImageJ2 software v 1.54f (Fiji) (National Institute of Mental Health). For each experimental variant, at least four samples were analyzed. In each sample, at least 200 cells were analyzed. The method is described in more detail in previous work [79,80].

### 2.10. Statistical Processing

The normality of sample distributions was tested using the Shapiro–Wilk test. The data obtained were presented as mean values ± standard error (SE). Statistical hypotheses were tested using the one-way Kruskal–Wallis rank analysis of variance (ANOVA) with Dunn’s test method or the Mann–Whitney U test. Differences were considered statistically significant when the significance level *p* < 0.05 was reached. At least 3 independent measurements were performed in each experiment. The exact sample sizes are given in the legends to the corresponding figures.

## 3. Results

### 3.1. The Physicochemical Properties of the Obtained Nanoparticles

**Figure 3:**
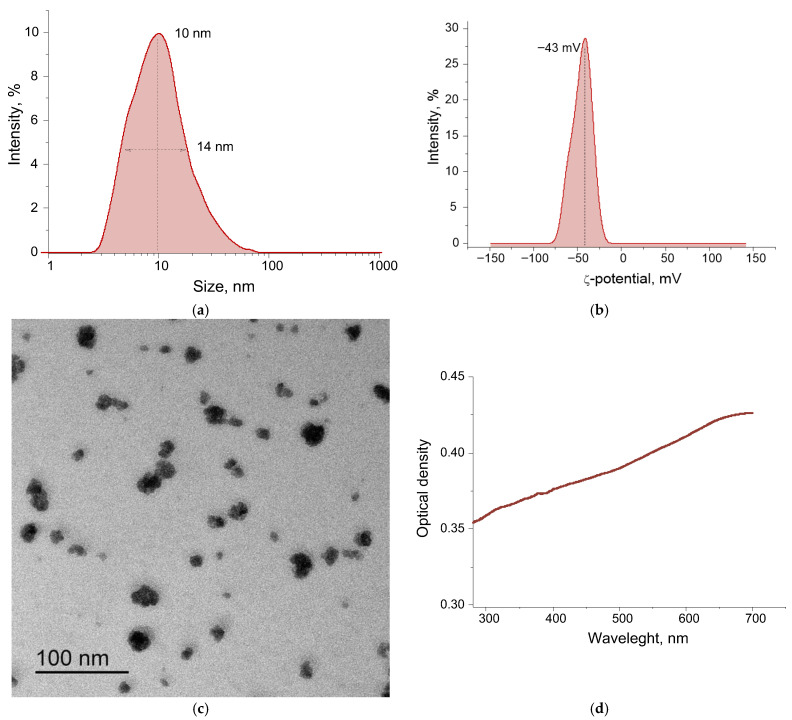
Physicochemical characteristics of TeNPs: distribution of NPs by size (**a**) and ζ-potential (**b**), TEM micrograph of a group of NPs (**c**), absorption spectrum of a suspension of TeNPs (**d**).

The average size of TeNPs measured by DLS is ~10 nm (Figure 3a). The distribution of TeNPs is monomodal. The half-width at the half-maximum of the distribution is 14 nm. It is shown that the peak of the ζ-potential distribution of TeNPs in an aqueous colloid is at −43 mV (Figure 3b). The ζ-potential value > 30 mV (modulo) indicates high stability of the colloid of the obtained TeNPs in water [81]. The absorption spectrum of the colloidal solution of nanoparticles in the range of 300–700 nm is presented below (Figure 3). The absorption of the TeNP colloid increases with increasing radiation wavelength and is greatest in the long-wavelength region of the spectrum. The region with an increased absorption of 500–700 nm corresponds to the literature data for TeNPs, while the observed relatively low absorption intensity is characteristic of spherical shapes [82]. Before introducing the obtained TeNPs into the polymer matrix, they were transferred to acetone (see above). In acetone, a change in the ζ-potential from −43 mV to −72 mV and an increase in the hydrodynamic radius from 15 to 208 nm were recorded.

### 3.2. Physicochemical Properties of the Obtained MPR+TeNP Composites

**Figure 4:**
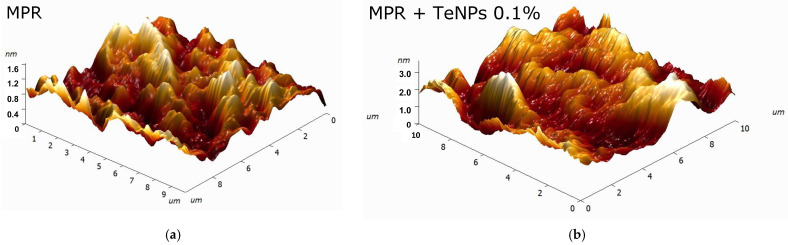
Three-dimensional reconstructions of MPR (**a**) or MPR+TeNPs 0.1% (**b**) surfaces obtained by AFM.

The surface microrelief of MPR and nanocomposites with TeNPs was assessed by AFM (Figure 4). The original polymer without the addition of NPs has a smooth surface (Figure 4a) without cracks and breaks. The size of the irregularities does not exceed 0.8 nm. The addition of TeNPs (even for the highest of the studied concentrations of 0.1%) did not affect the surface microrelief (Figure 4b). The total surface roughness did not exceed 2 nm with an average TeNP size of >10 nm (Figure 3a,c). It can be concluded that all NPs are enclosed in the polymer surface or immersed deeply enough so as not to change the microrelief of the surface of the resulting material.

**Figure 5:**
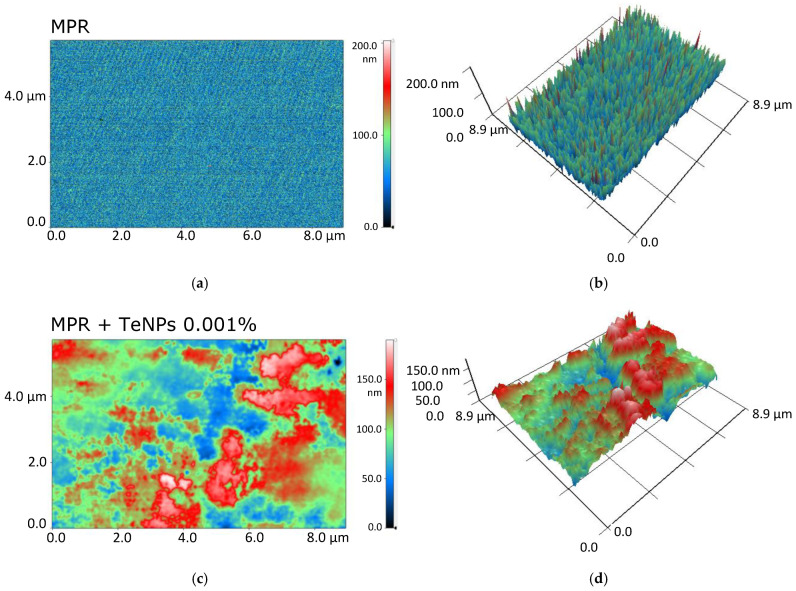

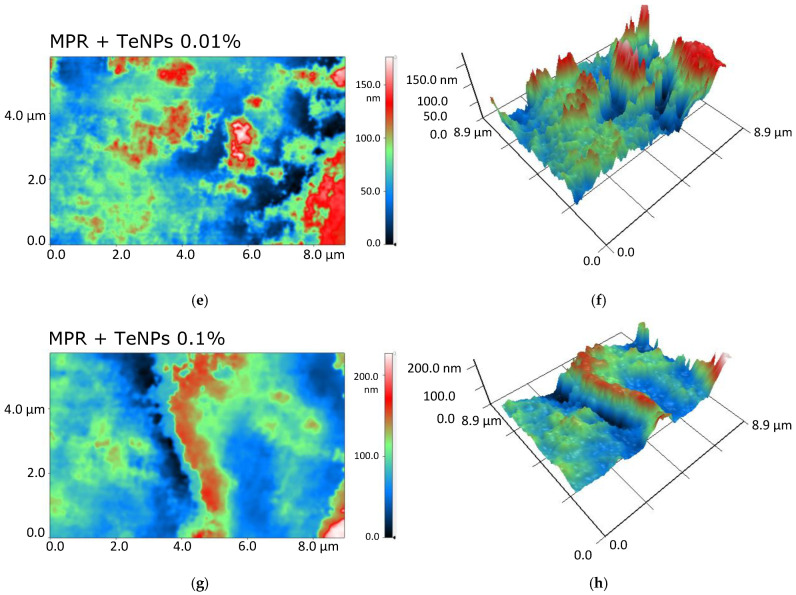
Examples of MIM micrographs of MPR without TeNPs (**a**,**b**) or MPR+TeNPsMPR+TeNPs with 0.001% (**c**,**d**), 0.01% (**e**,**f**), and 0.1% (**g**,**h**) TeNPs. The primary data (**a**,**c**,**e**,**g**) and 3D reconstructions of material sections measuring 8.9 × 8.9 μm (**b**,**d**,**f**,**h**). The phase difference (indicated on axis z) of the transmitted radiation is shown in color (red is the maximum value, blue is the minimum).

Since TeNPs inside the polymer were not detected by AFM, we applied the MIM method to search for and evaluate the distribution of TeNPs inside the polymer matrix (Figure 5). For the composite material based on MPR+TeNPs, a wide variety of sizes of optical inhomogeneities and a pronounced dependence of their sizes on the concentration of introduced TeNPs were observed (Figure 5). With an increase in the concentration of TeNPs from 0.001 to 0.1, the maximum dimensions of the optical inhomogeneities increased from 1–2 μm to 2 μm in diameter and >8 μm in length. The number of small inhomogeneities <0.2 μm decreased with an increase in the concentration of TeNPs.

**Figure 6:**
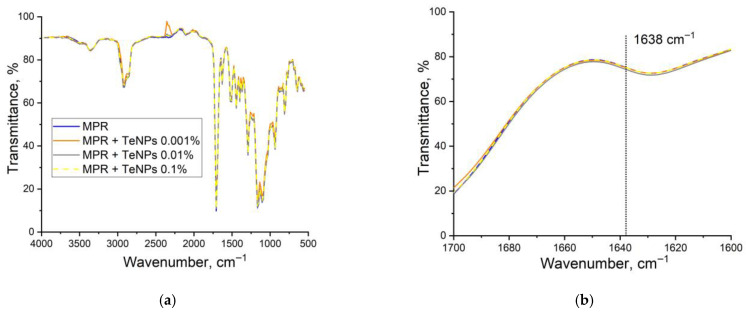
FTIR transmittance spectrum of samples of samples printed from MPR without NPs (blue), MPR+TeNPs 0.001% (orange), MPR+TeNPs 0.01% (grey), or MPR+TeNPs 0.1% (yellow) (*v*/*v*). The absorption in regions 500–4000 cm^−1^ (**a**) and 1500–1600 cm^−1^ (**b**) are shown.

The averaged FTIR transmission spectra for the obtained composite samples with TeNPs in three concentrations are given below (Figure 6). The inset in Figure 6 shows an enlarged region of the spectra with highlighted lines at 1638 cm^−1^, corresponding to the stretching vibrations of the C=C double bonds of methacrylates [79]. In addition, other characteristic absorption bands are listed in Table 1.

The spectra of the MPR+TeNPs nanocomposite materials are very similar in shape to that of the pure polymer. Therefore, the presence of TeNPs at concentrations of 0.001–0.1% does not cause significant changes in the structure or composition of the polymer.

Methacrylate resin is composed of monomers and oligomers of methacrylates with varying molecular chain lengths, as well as photoinitiators that participate in the polymerization. Polymerization of methacrylates occurs through the breaking of the carbon–carbon double bond in the methacrylate group. Consequently, the degree of polymerization of methacrylate resins can be estimated by analyzing the spectral band at 1638 cm^−1^, which corresponds to C=C double bonds [80]. One of the major challenges associated with methacrylate materials is achieving a high degree of polymerization of the initial components.

**Figure 7:**
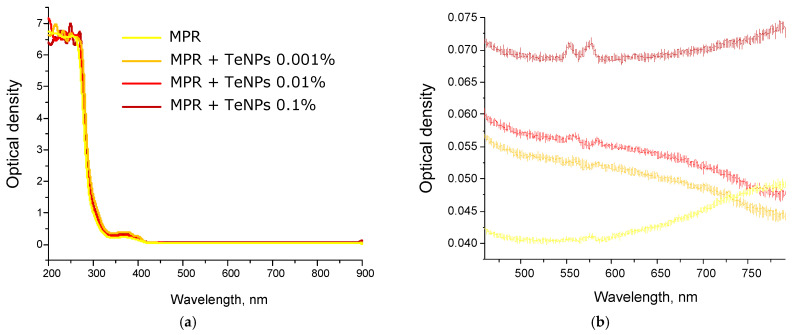
UV-vis absorption spectra of MPR or composite material samples with different TeNP content in region 200–900 nm (**a**) or only the visible region of 450–700 nm (**b**).

Examples of UV-vis absorption spectra are shown below (Figure 7). The spectra clearly show a broad absorption region of 200–300 nm, an absorption peak of 300–450 nm, as well as peaks of 545 and 555 nm (Figure 7b). MPR contains photoinitiators that allow photopolymerization of the material to obtain PMA. The spectrum region of 350–400 nm refers to the absorption of radiation by these photoinitiators. In general, it can be noted that the integration of NPs of all types does not significantly affect the optical properties of the polymethacrylate matrix in the visible range of the spectrum. All MPR+TeNP composite films have a transparency range from 450 to 800 nm. The absorption peaks of 545 and 555 nm are close to one of the absorption peaks of Te 530–540 nm [83,84]. The remaining absorption peaks of Te 255, 305, and 376 nm are masked by the absorption of the polymer matrix.

### 3.3. Jacobs Working Curves for Photopolymer Compositions and Study of Physical and Mechanical Properties

**Figure 8:**
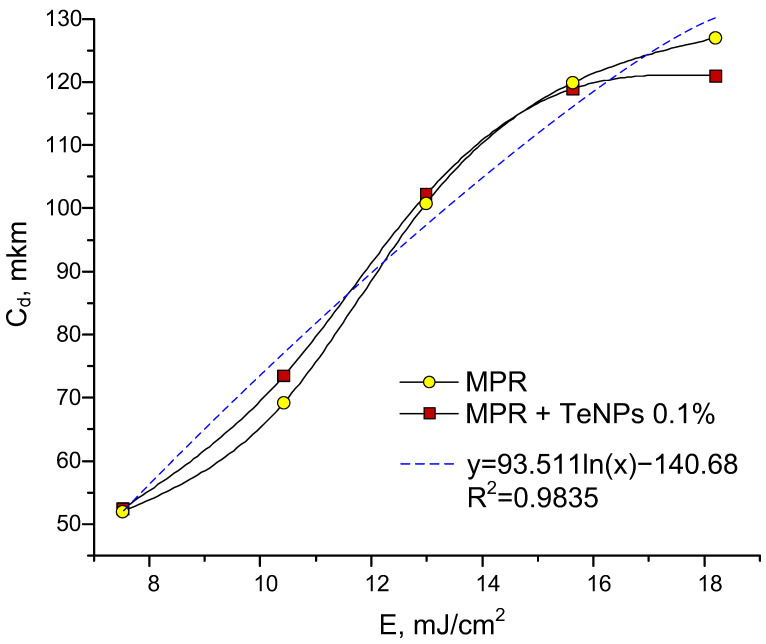
Jacobs working curves curves for MPR (gray dots) and MPR+TeNPs 0.1% (burgundy dots). The approximation for both graphs is shown by the dotted blue line. The regression equation and R^2^ for it are presented in the figure on the lower right.

The introduction TeNPs in PMR had virtually no effect on the reactivity of the resin, which can be easily described by Jacobs working curves, which represent the dependence of the thickness of the cured layer on the energy applied to the system [85,86]. At the maximum nanoparticle content of 0.1%, a coincidence of the curves with the unfilled photopolymer was observed (Figure 8).

This is explained by the fact that tellurium nanoparticles practically do not absorb at 405 nm, so they only make changes to the rheological properties of the composition. Based on the data obtained, the main characteristics of the compositions were determined, such as the critical energy of photopolymerization E_c_. and the penetration depth, D_p_. E_c_ is the energy that must be applied to start the photopolymerization process. These values are equal for all samples at 4.5 mJ/cm^2^ and 90 μm, for E_c_ and D_p_, respectively.

**Figure 9:**
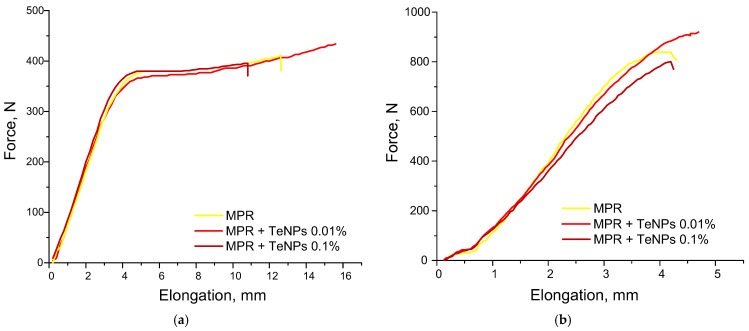
Mechanical properties assay of MPR without NPs (yellow line), MPR + TeNPs 0.01% (red line), and MPR + TeNPs 0.1% (burgundy line) for greenbody (**a**) and postcured (**b**) states.

The study of the physical and mechanical properties of the compositions was carried out immediately after printing (greenbody state) and post-processing (postcured state). The study of tensile strength is an important factor, since in the greenbody state, this parameter affects the possible lifting speeds of the printing platform, and the postcured state determines the final physical and mechanical properties. The elongation–force dependencies of control MPR and MPR+TeNPs 0.01–0.1% are shown below (Figure 9). These materials were selected based on FTIR data.

Greenbody MPR and MPR+TeNPs 0.1% have an equal tensile strength, Young’s modulus, and elongation at break: 27 MPa, 300 MPa, and 25%, respectively (Figure 9a). MPR+TeNPs 0.01% was observed to have a tendency towards increases in tensile strength and elongation at break compared to clear MPR.

Postcured MPR without dopant has a tensile strength of 60 MPa, Young’s modulus of 750 MPa, and elongation at break of 10% (Figure 9b). The addition of TeNPs 0.01% increased tensile strength to 74 MPa, Young’s modulus to 900 MPa, and elongation at break to 11%. These effects are associated with an increasing conversion of double bonds in the composition. It indicates a uniform TeNP distribution in the polymer matrix and the absence of defects/agglomerates in the printed products. In the case of MPR+TeNPs 0.1%, tensile strength, Young’s modulus, and elongation at break were decreased compared to MPR without NPs. These data are in accordance with the obtained FTIR spectra of MPR+TeNP composites (Figure 6).

### 3.4. Generation of ROS, 8-Oxoguanine, and LRPSs in the Presence of MPR+TeNP Composites

**Figure 10:**
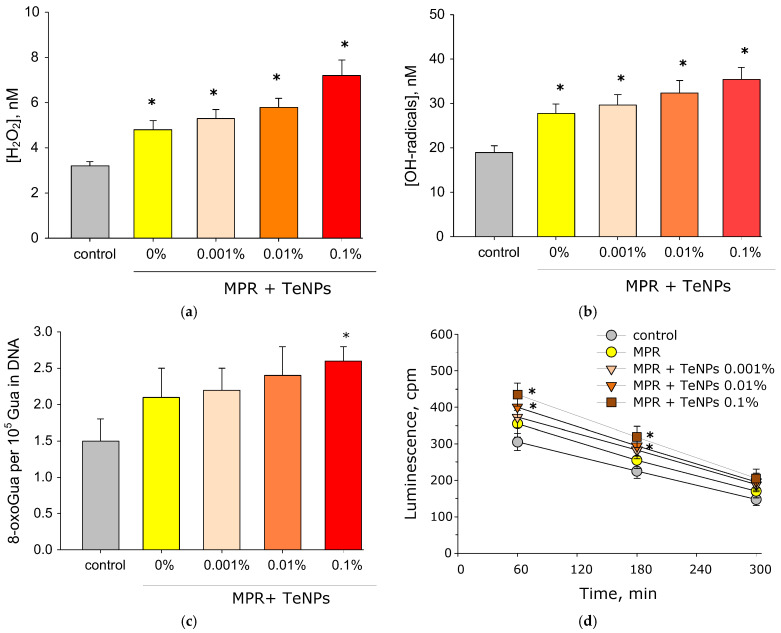
Evaluation of the generation of reactive species in the presence of the MPR+TeNP composite material with different dopant concentrations, MPR without NPs, and aqueous solutions without a sample (control). Generation of hydrogen peroxide after 3 h (**a**) and OH- radicals after 2 h (**b**) of incubation, 8-oxaguanine after 3 h, and (**c**) LRPSs after 2 h (**d**) of incubation with samples. Data are presented as mean values ± SE. *—*p* < 0.05 vs. control, Mann–Whitney U test (*n* = 3). On all panels the colors correspond by: gray—control, yellow—MPR without TeNPs, light orange—MPR+TeNPs 0.001%, dark orange—MPR+TeNPs 0.01%, and red—MPR+TeNPs 0.01%.

The results of the evaluation of the generation of reactive species are presented below (Figure 10). MPR without NPs and nanocomposite materials MPR+TeNPs 0.001 and 0.01% increased ROS generation. MPR without NPs increased H_2_O_2_ generation by ~70% relative to the control. MPR+TeNPs 0.001, 0.01, and 0.1% increased H_2_O_2_ generation by 1.8, 2.0 and 2.5 times compared to the control, respectively (Figure 10a). In the case of the OH- radicals, a similar picture was observed, but the increase in generation was approximately two times weaker compared to H_2_O_2_ (Figure 10b). MPR did not cause the generation of 8-oxoguanine. The addition of TeNPs to the polymer at a concentration of 0.001 or 0.01% also did not statistically significantly change the generation of 8-oxoguanine (Figure 10c). The composite material MPR+TeNPs 0.1% increased the generation of 8-oxoguanine by almost two times compared to the control. MPR+TeNPs 0.01% and 0.1% increased the generation of LRPSs compared to the control after 60 and 180 min of incubation by ~50% (Figure 10d). The remaining samples did not affect the generation of reactive forms of proteins.

### 3.5. Antibacterial Activity of MPR+TeNP Composites

**Figure 11:**
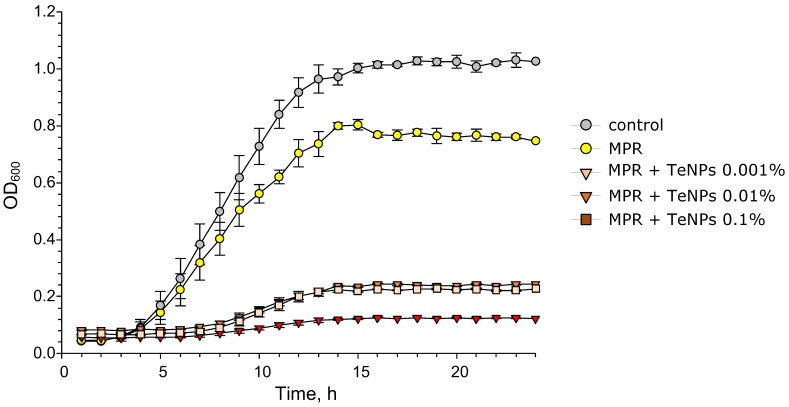
Growth curves of *E. coli* in LB broth suspension in the presence of MPR or MPR+TeNPs 0.001, 0.01, and 0.1%. Data are presented as mean values ± SE (*n* = 6).

Screening of antibacterial activity was performed using the growth curves of *E. coli* in LB broth suspension for 24 h in the control and in the presence of composite materials. The growth curves of *E. coli* cultured on PMC+TeNP composite materials for 24 h are shown in Figure 11. The MPR polymer without NPs reduced the maximum number of bacteria in the stationary phase by ~20% compared to the control. However, MPR did not affect the long lag phase. The PLA+TeNP composite material extended the lag period by 5 h compared to the control and MPR. In addition, PLA+TeNPs significantly inhibited bacterial growth after 24 h of incubation compared to pure PLA. The decrease in the maximum OD_600_ depended on the amount of NPs added to the polymer and was ~80% for MPR+TeNPs 0.001–0.01 and ~95% for MPR+TeNPs 0.1%. Therefore, the material MPR+TeNPs has a pronounced bacteriostatic effect, which increases with increasing dopant concentration.

**Figure 12:**
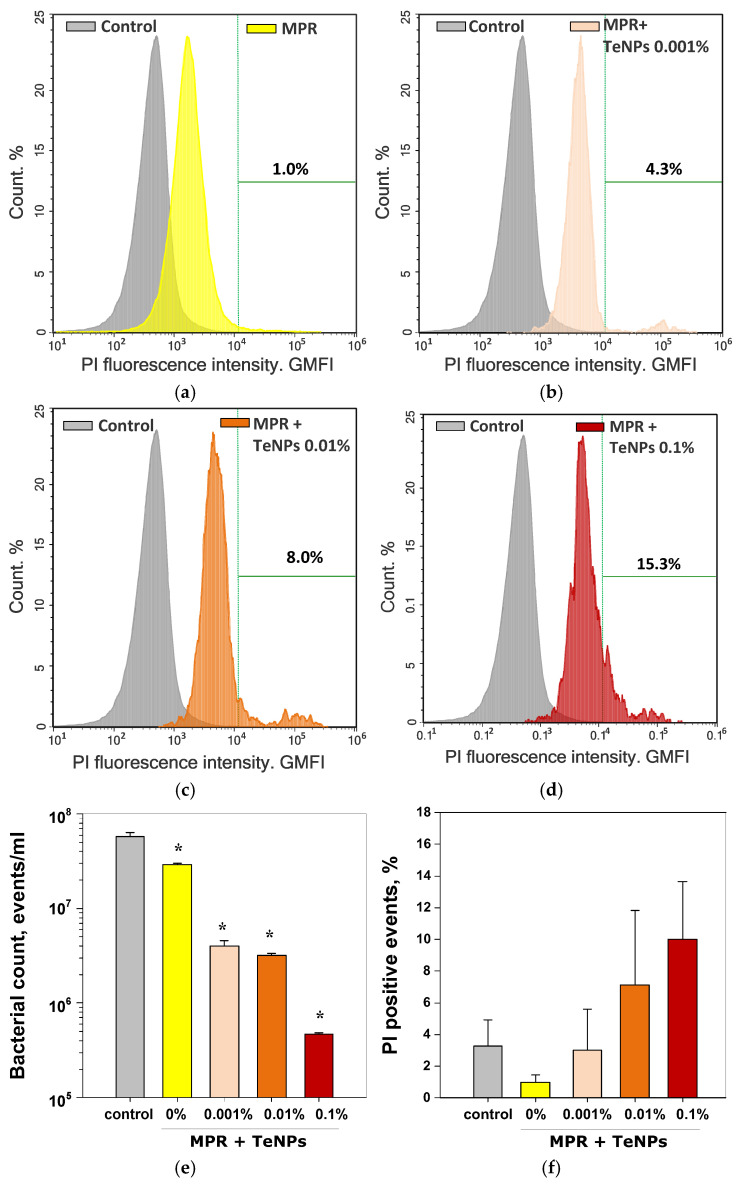
Evaluation of the antibacterial activity of nanocomposites against *E. coli* after 24 h of cultivation. Examples of histograms of bacterial cell distribution by PI staining (**a**–**d**) and averaged data on the number of cells/mL (**e**) and PI-positive events count (**f**). Data are presented as mean ± SE. *—*p* < 0.05 vs. control, Kruskal–Wallis ANOVA with Dunn’s test (*n* = 3).

To understand the mechanism of the antibacterial effect, we conducted a study of the bactericidal action of the composites using PI staining (penetrates dead cells) and an accurate cell count using flow cytometry to clarify the data obtained from the growth curves (Figure 12). We have discovered that the presence of MPR reduced the number of bacterial cells by ~two times compared to the control (Figure 12e). The decrease in the number of cells confirms the bacteriostatic effect of MPR, revealed by the growth curves. The addition of TeNPs enhanced the bacteriostatic effect of the composite material compared to the control and MPR. The magnitude of the effect depended on the concentration of TeNPs introduced into the composite. The presence of MPR+TeNPs 0.001% or 0.01% materials reduced the number of bacterial cells by ~17 times compared to the control, and MPR+TeNPs 0.1% reduced the number of cells by ~130 times. A tendency towards an increase in the number of dead bacteria was observed in samples cultured in the presence of MPR+TeNPs 0.01 and 0.1% Figure 12a–d) compared to the control. However, this trend did not reach statistical significance.

Therefore, the obtained composite materials exhibit antibacterial activity through the bacteriostatic effect.

### 3.6. Cytostatic Effect of MPR+TeNP Composites

**Figure 13:**
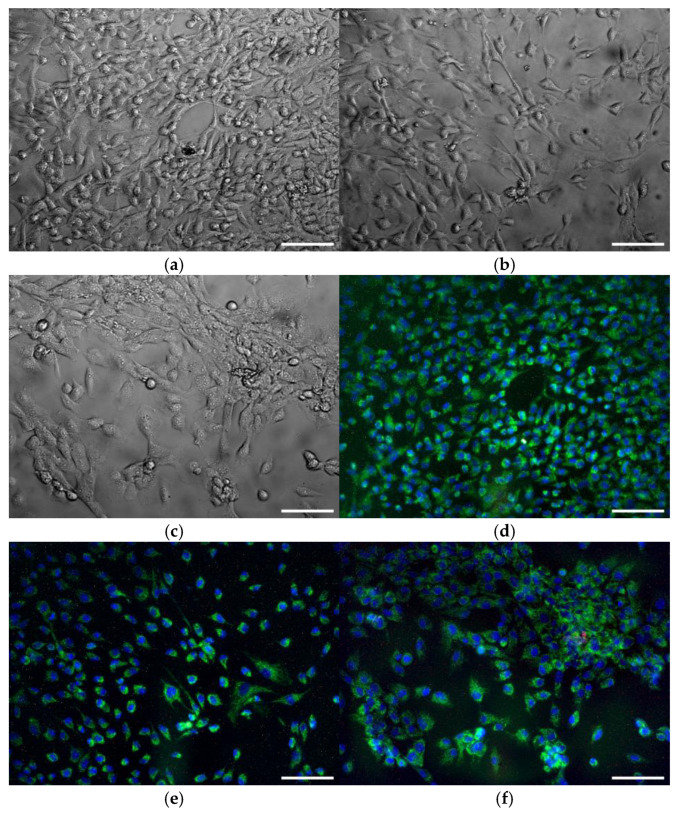

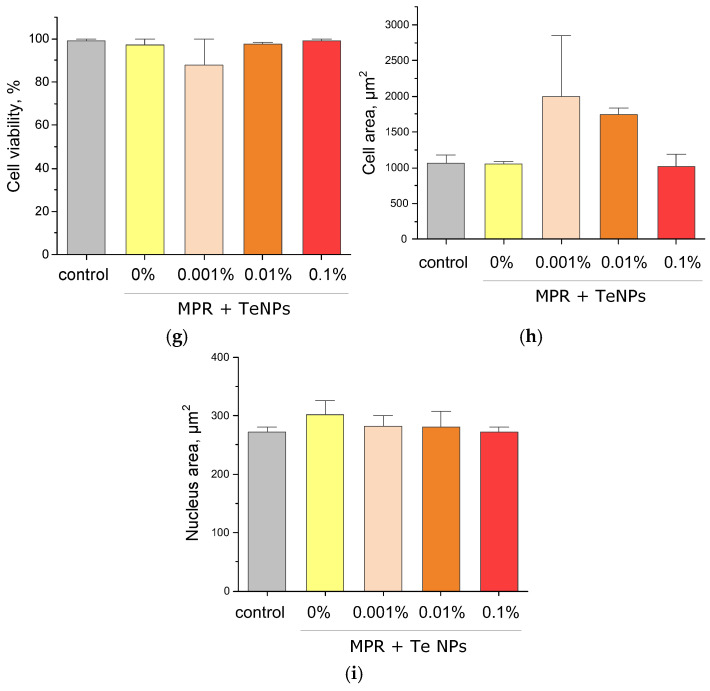
Cytotoxicity assessment nanocomposites. Examples of micrographs of HSF cell culture after 72 h of cultivation with control (**a**,**d**), in the presence of MPR (**b**,**e**) or MPR+TeNPs 0.1% composite (**c**,**f**). Shown are visible-light phase contrast images (**a**,**b**,**c**) and merged images of Hoechst (blue), Rhodamine 123 (green), and PI (red) (**d**,**e**,**f**). Average values of viability (**g**), single cell (**h**), and nucleus (**i**) areas after 72 h of culturing. Data are presented as means ± SE (*n* = 5). Scale bar is 100 μm. On panels g-i the colors correspond by: gray—control, yellow—MPR without TeNPs, light orange—MPR+TeNPs 0.001%, dark orange—MPR+TeNPs 0.01%, and red—MPR+TeNPs 0.01%.

The exposure of HSF cells over 72 h to the presence of MPR did not change cell morphology, cell areas, or nucleus areas compared to the control (Figure 13a,b,g–h). A trend towards increased nuclear areas was observed after culture with MPR, but it did not reach statistical significance. (Figure 13h). Fibroblast culture in the presence of MPR+TeNPs 0.001% resulted in a tendency toward decreased cell viability. A tendency toward increased cell area was observed in the presence of MPR+TeNPs 0.001% or MPR+TeNPs 0.01%. However, according to the results of the Kruskal–Wallis ANOVA with Dunn’s test method, none of the observed differences reached statistical significance. Therefore, MPR or MPR+TeNPs 0.001–0.1% nanocomposites did not cause significant cytotoxic effects on cell morphology (Figure 13a–f), nuclear areas, and viability (Figure 13g–h) during 72 h of exposure to HSF. Based on the results obtained, it can be concluded that composite materials do not have chronic cytotoxicity against human fibroblasts.

## 4. Discussion

Using the laser ablation method in water, we obtained TeNPs with monomodal distributions of size and ζ-potential (Figure 3). The sizes of tellurium nanoparticles were determined using two independent methods: dynamic light scattering (Figure 3a) and transmission electron microscopy (Figure 3c). Dynamic light scattering revealed that the colloid contains nanoparticles with characteristic sizes ranging from 3 to 60 nm, with nanoparticles around 10 nm being the most common. The TEM image clearly shows nanoparticles around 5 nm in size, with the largest nanoparticles being around 50 nm. The most common nanoparticle size is also around 10 nm. The ζ-potential value > 30 mV (modulo) indicates the high stability of the colloid of the obtained TeNPs in water [87]. After transfer to acetone for subsequent in situ polymerization, an increase in the ζ-potential from −43 mV to −72 mV was recorded, which indicates an additional increase in the stability of the NP colloid before the introduction of MPR monomers into the solution. Figure 3d shows the absorption spectrum of an aqueous colloid of tellurium nanoparticles. Overall, the spectral pattern is typical for similar tellurium colloids.

AFM results indicate that the TeNPs are almost completely embedded in the polymer matrix (Figure 4), and the MIM results allowed us to detect heterogeneities in the distribution of TeNPs in the MPR thickness (Figure 5). The formation of nanoparticle clusters in the thickness of a polymer matrix has been widely described in the literature, and the shape of the clusters can be determined by the nature of the polymer matrix or the characteristics of the doped nanoparticles (composition, shape, size, etc.) [88,89,90]. In the case of TeNPs, the concentration of the introduced particles significantly determines the size of the resulting clusters.

Polymerization of methacrylates occurs with the rupture of the carbon–carbon double bond in the methacrylate group. Consequently, the degree of polymerization of methacrylate resins can be estimated from the bands in the spectrum related to the C= C double bonds [91]. One of the important problems associated with methacrylate materials is the difficulty in achieving a high degree of polymerization of the original components. With incomplete polymerization, the resulting polymer contains monomers that have potential cyto and genotoxicity, which, in biomedical applications, can deeply penetrate tissues, cause allergic reactions, and increase the risk of pregnancy disorders [92,93,94,95]. Thus, it is important to reduce the toxicity of MPR by increasing the degree of polymerization. For this purpose, various polymerization and post-processing modes of polymers are selected [17]. However, some authors involved in the development of NP/polymer composites point out that the introduction of NPs can lead to additional cross-linking of polymer molecules. Therefore, the degree of polymerization can be increased in the presence of NPs [96,97,98]. The degree of polymerization of the composites obtained in this work was estimated by the intensity of the peaks in the region of 1611 and 1637 cm^−1^ in the IR Fourier absorption spectra of the materials. We found that the introduction of TeNPs into MPR dose-dependently changed the degree of photopolymerization of methacrylate. It is likely that the introduction of these TeNPs in higher concentrations affects the photopolymerization process, reducing the amount of polymerized monomer. In particular, TeNPs are capable of photocatalyzing the degradation of methylene blue [82]. It is possible that the photocatalytic properties of Te are also realized during the polymerization of methacrylate at low concentrations of TeNPs.

In addition to absorption in the visible region, Te can absorb in the UV region at ~376 nm [84]. It is possible that TeNPs at a concentration of 0.1% begin to absorb UV to such an extent that they begin to interfere with the polymerization of methacrylate. Finally, it cannot be completely denied that the presence of these NPs affects the intensity of the bands in the FTIR spectrum, and this method for determining the degree of polymerization from Fourier-IR spectra needs to be improved.

The addition of TeNPs 0.01–0.001% to MPR changes the mechanical properties of the polymer material depending on the TeNP concentration. TeNPs at a concentration of 0.01% lead to an increase in tensile strength, Young’s modulus, and elongation at break compared to MPR without a dopant. With an increase in the concentration of TeNPs by 0.1% in the polymer matrix, a decrease in all the studied characteristics was observed. The obtained data are consistent with the FTIR spectrometry data and may indicate changes in the TeNP-dependent degree of polymerization. Our data on the complex dependence of the degree of polymerization of nanocomposites on the concentration of added nanoparticles are consistent with those described in the literature [48,99].

We have found that nanocomposite materials including MPR+TeNPs have a pronounced bacteriostatic, but not bactericidal, effect. Given the potential biomedical applications, the question arises as to which mechanism of antibacterial activity is preferable: bacteriostatic or bactericidal? The destruction of bacterial cells inside the body leads to the induction of proinflammatory reactions involving innate immunity via TLR-dependent pathways and adaptive immunity involving T-, B-, and other cells [100,101]. The strength of the induced proinflammatory reaction depends on the number of dead bacterial cells. In mass bacteremia, the destruction of bacterial cells can lead to hyperactivation of the immune system, which is life-threatening [102,103,104]. Perhaps a bacteriostatic effect is more justified for biomedical purposes, since it will reduce the level of local inflammation.

Generation of ROS is one of the most discussed mechanisms for the antibacterial action of NPs [105,106,107,108,109]_._ H_2_O_2_ is one of the most stable ROS, and is capable of being transported across the membrane. OH- radicals are highly reactive, but are unable to penetrate cell membranes [110,111,112,113]. Generation of moderate amounts of ROS is an important part of the regulation of normal cell division, differentiation, and migration [114]. Excessive production of ROS and/or disruption of antioxidant system functioning can lead to the development of “oxidative stress”, leading to genotoxic effects, protein inactivation, and lipid peroxidation [115]. The above processes increase the risks of developing neoplasms, and mutagenesis, and accelerates the aging process of the organism [116,117,118]. MPR without NPs and MPR+TeNPs 0.001 and 0.01% materials increased the generation of ROS (H_2_O_2_ and OH- radicals). The intensity of hydrogen peroxide generation increased with increasing concentration of TeNPs. In the case of OH- radicals, a similar picture was observed, but the increase in generation was approximately two times weaker compared to H_2_O_2_ (Figure 10). It is worth noting that the generated concentrations of H_2_O_2_ and OH- radicals did not exceed tens of nM (Figure 10). These ROS concentrations are ≥3 orders of magnitude lower than those that can cause biological effects and ≥6 orders of magnitude lower than toxic ones [119,120], so it can be assumed that the obtained nanocomposite materials will not cause oxidative stress through ROS. Therefore, the bacteriostatic effect is probably due to other ROS-independent mechanisms.

In this work, we found that the composite material MPR+TeNPs 0.1% increased the generation of 8-oxoguanine to 2.6 per 10^5^ guanines in DNA. For human lymphocytes, the normal amount of 8-oxoguanine is 0.2–0.3 per 10^5^ guanines [121,122]. In pathologies associated with oxidative stress, its amount increases to 1.0–1.3 8-oxodG/10 ^5^ guanines [123]. In cancer cells, ~1.3 to ~10.7 8- oxodG /10^5^ guanines has been recorded [124,125]. The value we obtained is higher than normal, however, it should be taken into account that we assessed the generation of 8-oxoguanines in vitro. Active 8-oxoguanine repair systems exist in mammalian cells [126,127]. In addition, the “normal” amount of 8-oxoguanine depends on the cell type. For example, in intestinal cells, an amount of 1.6 8-oxoguanine per 10^5^ guanines is the normal [128]. Therefore, we can expect that the generation of 8-oxoguanine in the presence of the developed material in vivo will not exceed normal values. Our assumptions are confirmed by the absence of cytotoxicity for all obtained materials against human fibroblasts HSF during a three-day cultivation. The 8-oxoguanine reparation system in prokaryotes is simpler than in eukaryotes and has less perfect regulation [129]. In view of this, we believe that the bacteriostatic effect of the nanocomposite material may be realized through the induction of 8-oxoguanine generation.

**Figure 14:**
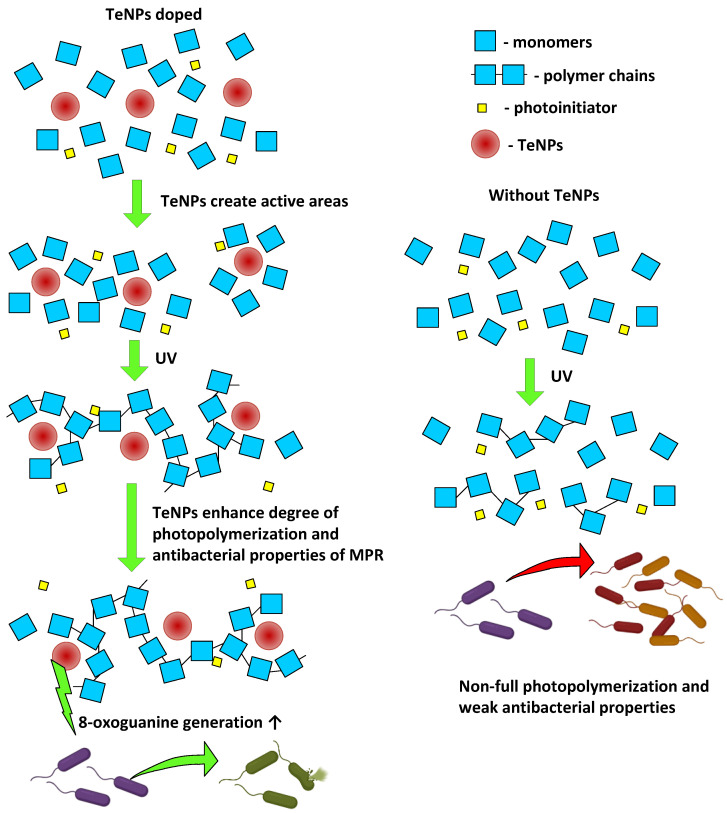
The influence on TeNPs on photopolymerization and antibacterial properties.

LRPSs are formed during protein modification, and LRPSs can promote new secondary free radical generation and lead to damage in DNA and other biomolecules [130,131,132,133,134,135]. The protection of cells against LRPSs is provided via ascorbate- and glutathione-dependent pathways, proteasomes system, etc. [136]. Eukaryotes have a more sophisticated antioxidant system, as with DNA repair [137,138]. Therefore, it can be expected that the generated LRPSs may exhibit greater toxicity against bacteria and lesser toxicity against eukaryotic cells. This assumption is supported by our data: *E. coli* growth curves and cytotoxicity assay HSF (Figure 13). Consequently, the bacteriostatic effect of MPR+TeNPs is realized through the induction of biodamage to macromolecules (proteins and DNA), in which TeNPs play a leading role (MPR in its pure form does not activate the generation of 8-oxoguanine and LRPSs). The general scheme of influence of TeNP doping on MPR photopolymerization and antibacterial properties is shown above (Figure 14).

The absence of a cytostatic effect can be explained by the more perfect structure of antioxidant and reparation systems in eukaryotes compared to prokaryotes. We observed a tendency towards HSF viability in presence of MPR, which disappeared in presence of MPR+TeNPs 0.01–0.1%. This result may potentially be explained by the antioxidant activity of TeNPs in vitro. TeNPs and SeNPs can reduce PMA-induced ROS generation by innate immune RAW264.7 cells or THP-1 cells via signal pathway modulation [139].

## 5. Conclusions

In this paper, the in situ polymerization method was used to obtain a set of composite polymeric materials based on methacrylate photopolymer resin and Te nanoparticles introduced at different concentrations (MPR+TeNPs 0.001, 0.01, or 0.1% *v*/*v*). Doping TeNPs changed the mechanical properties of the specified materials, altered the degree of polymerization, and improved the antibacterial activity of MPR. The mechanism of antibacterial action of MPR+TeNPs probably consists of enhancing the generation of 8-oxoguanine and long-lived reactive protein species in aqueous solutions. The synthesized material did not have cytotoxicity with respect to human fibroblasts. The most optimal concentration of the introduced MPR+TeNPs is 0.01%, so in this case the balance between a high degree of polymerization, increased mechanical properties, high bacteriostatic effects, and the absence of cytotoxicity is maintained.

## Figures and Tables

**Table 1:** IR absorption bands of MPR.

Absorption Band, cm^−1^	Assignment
3400–3200	O-H stretching vibrations
2950–2800	C-H stretching vibrations
1713	C=O stretching vibrations
881	=CH deformation vibrations

## Data Availability

The data presented in this study are available on request from the corresponding author due to order of the Head of the Biophotonics Center of the GPI RAS (on the basis of an agreement with the grant-giving organization).

## Abbreviations

The following abbreviations are used in this manuscript:

AFM	Atomic force microscopy
ANOVA	Analysis of variance
BPP	Bath photopolymerization
DLS	Dynamic light scattering
ELISA	Enzyme-linked immunosorbent assay
FTIR	Fourier-transform infrared spectroscopy
HSF	Human spleen fibroblasts
LRPSs	Long-lived reactive proteins species
MIM	Modulation interference microscopy
MPR	Methacrylate photopolymer resin
NPs	Nanoparticles
PI	Propidium iodide
PMMA	Poly(methyl methacrylate)
ROS	Reactive oxygen species
SeNPs	Selenium nanoparticles
TEM	Transmission electron microscopy
TeNPs	Tellurium nanoparticles
UV	Ultraviolet

## References

[1] Puzatova A., Shakor P., Laghi V., Dmitrieva M. (2022). Large-Scale 3D Printing for Construction Application by Means of Robotic Arm and Gantry 3D Printer: A Review. Buildings.

[2] Konnikov E.A., Konnikova O.A., Rodionov D.G. (2019). Impact of 3D-Printing Technologies on the Transformation of Industrial Production in the Arctic Zone. Resources.

[3] Girskas G., Kligys M. (2025). 3D Concrete Printing Review: Equipment, Materials, Mix Design, and Properties. Buildings.

[4] Aghajani M., Garshasbi H.R., Naghib S.M., Mozafari M.R. (2025). 3D Printing of Hydrogel Polysaccharides for Biomedical Applications: A Review. Biomedicines.

[5] Kwon S., Hwang D. (2025). Understanding and Resolving 3D Printing Challenges: A Systematic Literature Review. Processes.

[6] Sun K., Peng X., Gan Z., Chen W., Li X., Gong T., Xiao P. (2022). 3D Printing/Vat Photopolymerization of Photopolymers Activated by Novel Organic Dyes as Photoinitiators. Catalysts.

[7] Timofticiuc I.-A., Călinescu O., Iftime A., Dragosloveanu S., Caruntu A., Scheau A.-E., Badarau I.A., Didilescu A.C., Caruntu C., Scheau C. (2023). Biomaterials Adapted to Vat Photopolymerization in 3D Printing: Characteristics and Medical Applications. J. Funct. Biomater..

[8] Andjela L., Abdurahmanovich V.M., Vladimirovna S.N., Mikhailovna G.I., Yurievich D.D., Alekseevna M.Y. (2022). A review on Vat Photopolymerization 3D-printing processes for dental application. Dent. Mater..

[9] Dutta A., Rajasekaran R., Ray P.G., Seesala V.S., Dogra N., Ghorai S.K., Ojha A., Mukherjee K., Gupta S., Chattopadhyay S. (2023). Influence of surface engineering on 3D printed Ti lattice structure towards enhanced tissue integration: An in vitro and in vivo study. Talanta Open.

[10] Ghorai S.K., Maji S., Subramanian B., Maiti T.K., Chattopadhyay S. (2019). Coining attributes of ultra-low concentration graphene oxide and spermine: An approach for high strength, anti-microbial and osteoconductive nanohybrid scaffold for bone tissue regeneration. Carbon.

[11] Hata K., Ikeda H., Nagamatsu Y., Masaki C., Hosokawa R., Shimizu H. (2021). Development of Dental Poly(methyl methacrylate)-Based Resin for Stereolithography Additive Manufacturing. Polymers.

[12] Khatoon S., Khandelwal A., Raj A., Ahmad G. (2023). Fabrication of FFF 3D-printed surfaces for PMMA-based biomedical device employing the pre-processing optimization to eliminate the post-processing steps. Prog. Addit. Manuf..

[13] Ramanathan S., Lin Y.-C., Thirumurugan S., Hu C.-C., Duann Y.-F., Chung R.-J. (2024). Poly(methyl methacrylate) in Orthopedics: Strategies, Challenges, and Prospects in Bone Tissue Engineering. Polymers.

[14] Thirugnanasamabandam A., Nallamuthu R., Renjit M., Gnanasagaran C.L. (2024). 3D-printed PLA/PMMA polymer composites: A consolidated feasible characteristic investigation for dental applications. Polym. Eng. Sci..

[15] Yarikov A., Gorbatov R., Stolyarov I., Smirnov I., Fraerman A., Sosnin A., Perlmutter O. (2021). Application of additive 3D printing technologies in traumatology/orthopedics and neurosurgery. Vrach.

[16] Aftab M., Ikram S., Ullah M., Khan N., Naeem M., Khan M.A., Bakhtiyor o’g’li R.B., Qizi K.S.S., Erkinjon Ugli O.O., Abdurasulovna B.M. (2025). Recent Trends and Future Directions in 3D Printing of Biocompatible Polymers. J. Manuf. Mater. Process..

[17] Filimonova E.A., Lozovaya A.V., Prosyankin E.E., Mustafina A.R., Chapala P. (2024). Thermal treatment influence on optical properties of 3D printed objects by vat photopolymerization. Prog. Addit. Manuf..

[18] Zhakeyev A., Zhang L., Xuan J. (2020). Photoactive resin formulations and composites for optical 3D and 4D printing of functional materials and devices. 3D and 4D Printing of Polymer Nanocomposite Materials.

[19] Arossi G.A., Abdou N.A., Hung B., Garcia I.M., Zimmer R., Melo M.A. (2024). Safety of 3D-Printed Acrylic Resins for Prosthodontic Appliances: A Comprehensive Cytotoxicity Review. Appl. Sci..

[20] Brooks A.K., Yadavalli V.K. (2024). Post-Print Processing to Minimize Cytotoxicity of 3D-Printed Photopolymer Resins for Biomedical Applications. J. Appl. Polym. Sci..

[21] Azlin M., Ilyas R., Zuhri M., Sapuan S., Harussani M., Sharma S., Nordin A., Nurazzi N., Afiqah A. (2022). 3D Printing and Shaping Polymers, Composites, and Nanocomposites: A Review. Polymers.

[22] Darbandi K.R., Amin B.K. (2024). Innovation and Evaluations of 3D Printing Resins Modified with Zirconia Nanoparticles and Silver Nanoparticle-Immobilized Halloysite Nanotubes for Dental Restoration. Coatings.

[23] Jain K., Shukla R., Yadav A., Ujjwal R.R., Flora S.J.S. (2021). 3D Printing in Development of Nanomedicines. Nanomaterials.

[24] Luo X., Cheng H., Wu X. (2023). Nanomaterials Reinforced Polymer Filament for Fused Deposition Modeling: A State-of-the-Art Review. Polymers.

[25] Shah M., Ullah A., Azher K., Ur Rehman A., Akturk N., Juan W., Tüfekci C.S., Salamci M.U. (2023). The Influence of Nanoparticle Dispersions on Mechanical and Thermal Properties of Polymer Nanocomposites Using SLA 3D Printing. Crystals.

[26] Zhang N., Wang Z., Zhao Z., Zhang D., Feng J., Yu L., Lin Z., Guo Q., Huang J., Mao J. (2025). 3D printing of micro-nano devices and their applications. Microsyst. Nanoeng..

[27] Li P., Yin R., Cheng J., Lin J. (2023). Bacterial Biofilm Formation on Biomaterials and Approaches to Its Treatment and Prevention. Int. J. Mol. Sci..

[28] Halawa E.M., Fadel M., Al-Rabia M.W., Behairy A., Nouh N.A., Abdo M., Olga R., Fericean L., Atwa A.M., El-Nablaway M. (2024). Antibiotic action and resistance: Updated review of mechanisms, spread, influencing factors, and alternative approaches for combating resistance. Front. Pharmacol..

[29] Evstigneeva S.S., Chumakov D.S., Tumskiy R.S., Khlebtsov B.N., Khlebtsov N.G. (2023). Detection and imaging of bacterial biofilms with glutathione-stabilized gold nanoclusters. Talanta.

[30] Nastulyavichus A.A., Tolordava E.R., Ulturgasheva E.V., Shelygina S.N., Babina S.P., Saraeva I.N., Kudryashov S.I. (2025). Study of Laser Transfer Regimes to Increase the Efficiency of Application of Antibacterial Silver Nanoparticles. Bull. Lebedev Phys. Inst..

[31] Gudkov S.V., Serov D.A., Astashev M.E., Semenova A.A., Lisitsyn A.B. (2022). Ag_2_O Nanoparticles as a Candidate for Antimicrobial Compounds of the New Generation. Pharmaceuticals.

[32] Bakina O., Svarovskaya N., Ivanova L., Glazkova E., Rodkevich N., Evstigneev V., Evstigneev M., Mosunov A., Lerner M. (2023). New PMMA-Based Hydroxyapatite/ZnFe2O4/ZnO Composite with Antibacterial Performance and Low Toxicity. Biomimetics.

[33] Badaraev A.D., Lerner M.I., Bakina O.V., Sidelev D.V., Tran T.-H., Krinitcyn M.G., Malashicheva A.B., Cherempey E.G., Slepchenko G.B., Kozelskaya A.I. (2023). Antibacterial Activity and Cytocompatibility of Electrospun PLGA Scaffolds Surface-Modified by Pulsed DC Magnetron Co-Sputtering of Copper and Titanium. Pharmaceutics.

[34] Kozelskaya A.I., Verzunova K.N., Akimchenko I.O., Frueh J., Petrov V.I., Slepchenko G.B., Bakina O.V., Lerner M.I., Brizhan L.K., Davydov D.V. (2023). Antibacterial Calcium Phosphate Coatings for Biomedical Applications Fabricated via Micro-Arc Oxidation. Biomimetics.

[35] Parmar A., Kaur G., Kapil S., Sharma V., Sachar S., Sandhir R., Sharma S. (2019). Green chemistry mediated synthesis of PLGA-Silver nanocomposites for antibacterial synergy: Introspection of formulation parameters on structural and bactericidal aspects. React. Funct. Polym..

[36] Smirnova V.V., Chausov D.N., Serov D.A., Kozlov V.A., Ivashkin P.I., Pishchalnikov R.Y., Uvarov O.V., Vedunova M.V., Semenova A.A., Lisitsyn A.B. (2021). A Novel Biodegradable Composite Polymer Material Based on PLGA and Silver Oxide Nanoparticles with Unique Physicochemical Properties and Biocompatibility with Mammalian Cells. Materials.

[37] Tolordava E.R., Nastulyavichus A.A., Ulturgasheva E.V., Shelygina S.N., Saraeva I.N., Kudryashov S.I. (2025). Antibacterial Activity of Metal Nanoparticles in an Ex Vivo Wound Infection Model. Mol. Genet. Microbiol. Virol..

[38] Khlebtsov B.N. (2023). Functional Nanoparticles: Synthesis and Practical Applications. Colloid J..

[39] Han H.-W., Patel K.D., Kwak J.-H., Jun S.-K., Jang T.-S., Lee S.-H., Knowles J.C., Kim H.-W., Lee H.-H., Lee J.-H. (2021). Selenium Nanoparticles as Candidates for Antibacterial Substitutes and Supplements against Multidrug-Resistant Bacteria. Biomolecules.

[40] Lesnichaya M., Perfileva A., Nozhkina O., Gazizova A., Graskova I. (2022). Synthesis, toxicity evaluation and determination of possible mechanisms of antimicrobial effect of arabinogalactane-capped selenium nanoparticles. J. Trace Elem. Med. Biol..

[41] Huang T., Holden J.A., Heath D.E., O’Brien-Simpson N.M., O’Connor A.J. (2019). Engineering highly effective antimicrobial selenium nanoparticles through control of particle size. Nanoscale.

[42] Alhadrami H.A., Shoudri R.A.M. (2020). Titanium Oxide (TiO_2_) Nanoparticles for Treatment of Wound Infection.

[43] Modi S., Yadav V.K., Choudhary N., Alswieleh A.M., Sharma A.K., Bhardwaj A.K., Khan S.H., Yadav K.K., Cheon J.-K., Jeon B.-H. (2022). Onion Peel Waste Mediated-Green Synthesis of Zinc Oxide Nanoparticles and Their Phytotoxicity on Mung Bean and Wheat Plant Growth. Materials.

[44] Tang A., Ren Q., Wu Y., Wu C., Cheng Y. (2022). Investigation into the Antibacterial Mechanism of Biogenic Tellurium Nanoparticles and Precursor Tellurite. Int. J. Mol. Sci..

[45] Abed R.N., Zainulabdeen K., Abdallh M., Yousif E., Rashad A.A., Jawad A.H. (2023). The optical properties behavior of modify poly(methyl methacrylate) nanocomposite thin films during solar energy absorption. J. Non-Cryst. Solids.

[46] Alkayal N.S., Al Ghamdi M.A. (2025). Cross-Linked Poly(methyl methacrylate) Nanocomposites’ Synthesis, Characterization, and Antibacterial Effects. Polymers.

[47] El-Bashir S., Althumairi N., Alzayed N. (2017). Durability and Mechanical Performance of PMMA/Stone Sludge Nanocomposites for Acrylic Solid Surface Applications. Polymers.

[48] Galant K., Turosz N., Chęcińska K., Chęciński M., Cholewa-Kowalska K., Karwan S., Chlubek D., Sikora M. (2024). Silver Nanoparticles (AgNPs) Incorporation into Polymethyl Methacrylate (PMMA) for Dental Appliance Fabrication: A Systematic Review and Meta-Analysis of Mechanical Properties. Int. J. Mol. Sci..

[49] Gao Y., Chen X., Zhang Y., Dong X.-H., Yu Q., Wang L. (2025). Moisture-Resistant, High-Performance Polarizing Films via Aligned PMMA/CNT Composite Fibers: A Scalable Electrospinning Approach. Molecules.

[50] Komeijani M., Bahri-Laleh N., Mirjafary Z., D’Alterio M.C., Rouhani M., Sakhaeinia H., Moghaddam A.H., Mirmohammadi S.A., Poater A. (2025). PLA/PMMA Reactive Blending in the Presence of MgO as an Exchange Reaction Catalyst. Polymers.

[51] Uçar E., Dogu M., Demirhan E., Krause B. (2023). PMMA/SWCNT Composites with Very Low Electrical Percolation Threshold by Direct Incorporation and Masterbatch Dilution and Characterization of Electrical and Thermoelectrical Properties. Nanomaterials.

[52] Ao B., Jiang H., Cai X., Liu D., Tu J., Shi X., Wang Y., He F., Lv J., Li J. (2024). Synthesis of Tellurium Nanoparticles Using Moringa oleifera Extract, and Their Antibacterial and Antibiofilm Effects against Bacterial Pathogens. Microorganisms.

[53] Sári D., Ferroudj A., Semsey D., El-Ramady H., Brevik E.C., Prokisch J. (2024). Tellurium and Nano-Tellurium: Medicine or Poison?. Nanomaterials.

[54] Vahidi H., Kobarfard F., Alizadeh A., Saravanan M., Barabadi H. (2021). Green nanotechnology-based tellurium nanoparticles: Exploration of their antioxidant, antibacterial, antifungal and cytotoxic potentials against cancerous and normal cells compared to potassium tellurite. Inorg. Chem. Commun..

[55] Zhu H., Fan L., Wang K., Liu H., Zhang J., Yan S. (2023). Progress in the Synthesis and Application of Tellurium Nanomaterials. Nanomaterials.

[56] Palomba M., Binetti S., Le Donne A., Coscia U., Ambrosone G., Carotenuto G. (2016). Tellurium-based nanocomposites for plastic electronic applications. AIP Conference Proceedings, Proceedings of the VIII International Conference on “Times of Polymers and Composites”: From Aerospace to Nanotechnology, Naples, Italy, 19–23 June 2016.

[57] Wang L., Cao W., Xu H. (2016). Tellurium-Containing Polymers: Towards Biomaterials and Optoelectronic Materials. ChemNanoMat.

[58] Yadav A.K., Mohammad N., Khanna P.K. (2023). Novel synthesis of polyaniline/tellurium (PANI/Te) nanocomposite and its EMI shielding behavior. Mater. Adv..

[59] Chen Y., Sathiyaseelan A., Zhang X., Jin Y., Wang M.-H. (2025). Preparation of antibacterial tellurium nanorod-incorporated thermosensitive pluronic F-127 hydrogels for wound healing applications. J. Drug Deliv. Sci. Technol..

[60] Hu J., Ran S., Huang Z., Liu Y., Hu H., Zhou Y., Ding X., Yin J., Zhang Y. (2024). Antibacterial tellurium-containing polycarbonate drug carriers to eliminate intratumor bacteria for synergetic chemotherapy against colorectal cancer. Acta Biomater..

[61] Matharu R.K., Charani Z., Ciric L., Illangakoon U.E., Edirisinghe M. (2018). Antimicrobial activity of tellurium-loaded polymeric fiber meshes. J. Appl. Polym. Sci..

[62] Bergoglio M., Najmi Z., Ferla F., Scalia A.C., Cochis A., Rimondini L., Vernè E., Sangermano M., Miola M. (2025). Tellurium-Doped Silanised Bioactive Glass–Chitosan Hydrogels: A Dual Action for Antimicrobial and Osteoconductive Platforms. Polymers.

[63] Huang Y.-C., Zeng Y.-J., Lin Y.-W., Tai H.-C., Don T.-M. (2023). In Situ Encapsulation of Camptothecin by Self-Assembly of Poly(acrylic acid)-b-Poly(N-Isopropylacrylamide) and Chitosan for Controlled Drug Delivery. Polymers.

[64] Jin J., Liu C., Tong H., Sun Y., Huang M., Ren G., Xie H. (2022). Encapsulation of EGCG by Zein-Gum Arabic Complex Nanoparticles and In Vitro Simulated Digestion of Complex Nanoparticles. Foods.

[65] Megahed S., Wutke N., Liu Y., Klapper M., Schulz F., Feliu N., Parak W.J. (2024). Encapsulation of Nanoparticles with Statistical Copolymers with Different Surface Charges and Analysis of Their Interactions with Proteins and Cells. Int. J. Mol. Sci..

[66] Sánchez A., Mejía S.P., Orozco J. (2020). Recent Advances in Polymeric Nanoparticle-Encapsulated Drugs against Intracellular Infections. Molecules.

[67] Varlamova E.G., Gudkov S.V., Turovsky E.A. (2025). Opposite Effects of Small and Large Diameter Selenium Nanoparticles on the Redox-Status and Survival of Cortical Cells in Toxic Models In Vitro. Biol. Trace Elem. Res..

[68] Turovsky E.A., Varlamova E.G., Rogachev V.V., Gudkov S.V. (2025). Tellurium Nanoparticles Produced by Laser Ablation Induce Selective Anticancer Effects via ROS-Mediated Apoptosis and Calcium Signaling Pathways: In Vitro Screening. Biochem. Biophys. Res. Commun..

[69] (2020). Biological Evaluation of Medical Devices. Part 18: Chemical Characterization of Medical Device Materials Within a Risk Management Process.

[70] (2023). Plastics—Determination of Charpy Impact Properties. Part 1: Non-Instrumented Impact Test.

[71] (2025). Plastics—Determination of Tensile Properties. Part 2: Test Conditions for Moulding and Extrusion Plastics.

[72] Simakin A.V., Burmistrov D.E., Baimler I.V., Gritsaeva A.V., Serov D.A., Astashev M.E., Chapala P., Validov S.Z., Yanbaev F.M., Gudkov S.V. (2025). TiO_2_ Nanoparticles Obtained by Laser Sintering When Added to Methacrylate Photopolymer Resin Improve Its Physicochemical Characteristics and Impart Antibacterial Properties. Inorganics.

[73] Burmistrov D.E., Serov D.A., Baimler I.V., Gritsaeva A.V., Chapala P., Simakin A.V., Astashev M.E., Karmanova E.E., Dubinin M.V., Nizameeva G.R. (2025). Polymethyl Methacrylate-like Photopolymer Resin with Titanium Metal Nanoparticles Is a Promising Material for Biomedical Applications. Polymers.

[74] (2022). Standard Test Method for Tensile Properties of Plastics.

[75] Sevostyanov M.A., Kolmakov A.G., Sergiyenko K.V., Kaplan M.A., Baikin A.S., Gudkov S.V. (2020). Mechanical, physical–chemical and biological properties of the new Ti–30Nb–13Ta–5Zr alloy. J. Mater. Sci..

[76] Chernikov A.V., Gudkov S.V., Shtarkman I.N., Bruskov V.I. (2007). Oxygen effect in heat-induced DNA damage. Biophysics.

[77] Ivanov V.E., Usacheva A.M., Chernikov A.V., Bruskov V.I., Gudkov S.V. (2017). Formation of long-lived reactive species of blood serum proteins induced by low-intensity irradiation of helium-neon laser and their involvement in the generation of reactive oxygen species. J. Photochem. Photobiol. B Biol..

[78] Gudkov S.V., Shtarkman I.N., Chernikov A.V., Usacheva A.M., Bruskov V.I. (2007). Guanosine and inosine (riboxin) eliminate the long-lived protein radicals induced X-ray radiation. Dokl. Biochem. Biophys..

[79] Sarimov R.M., Nagaev E.I., Matveyeva T.A., Binhi V.N., Burmistrov D.E., Serov D.A., Astashev M.E., Simakin A.V., Uvarov O.V., Khabatova V.V. (2022). Investigation of Aggregation and Disaggregation of Self-Assembling Nano-Sized Clusters Consisting of Individual Iron Oxide Nanoparticles upon Interaction with HEWL Protein Molecules. Nanomaterials.

[80] Rudenko Y., Kozlov V., Burmistrov D., Fedyakova N., Bermeshev M., Chapala P. (2025). Comprehensive investigation of phosphine oxide photoinitiators for vat photopolymerization. Prog. Addit. Manuf..

[81] Pochapski D.J., Carvalho dos Santos C., Leite G.W., Pulcinelli S.H., Santilli C.V. (2021). Zeta Potential and Colloidal Stability Predictions for Inorganic Nanoparticle Dispersions: Effects of Experimental Conditions and Electrokinetic Models on the Interpretation of Results. Langmuir.

[82] Wang H., Zou H., Wang C., Lv S., Jin Y., Hu H., Wang X., Chi Y., Yang X. (2023). Controllable Synthesis, Formation Mechanism, and Photocatalytic Activity of Tellurium with Various Nanostructures. Micromachines.

[83] Tsai H.-W., Yaghoubi A., Chan T.-C., Wang C.-C., Liu W.-T., Liao C.-N., Lu S.-Y., Chen L.-J., Chueh Y.-L. (2015). Electrochemical synthesis of ultrafast and gram-scale surfactant-free tellurium nanowires by gas–solid transformation and their applications as supercapacitor electrodes for p-doping of graphene transistors. Nanoscale.

[84] Grasser M.A., Pietsch T., Blasius J., Hollóczki O., Brunner E., Doert T., Ruck M. (2021). Coexistence of Tellurium Cations and Anions in Phosphonium-Based Ionic Liquids. Chem. A Eur. J..

[85] Rudenko Y., Prosyankin E., Mustafina A., Fuki M., Borisov R., Fedyakova N., Bermeshev M., Chapala P. (2025). Investigation of the Light Intensity and Temperature Influences on Double Bond Conversion in Resins for Vat Photopolymerization via Fourier Transform Infrared Spectroscopy. Macromol. Chem. Phys..

[86] Rudenko Y., Lozovaya A., Asanova L., Fedyakova N., Chapala P. (2023). Light intensity influence on critical energy and penetration depth for vat photopolymerization technology. Prog. Addit. Manuf..

[87] Dzhardimalieva G., Bondarenko L., Illés E., Tombácz E., Tropskaya N., Magomedov I., Orekhov A., Kydralieva K. (2021). Colloidal Stability of Silica-Modified Magnetite Nanoparticles: Comparison of Various Dispersion Techniques. Nanomaterials.

[88] Oberdisse J. (2006). Aggregation of colloidal nanoparticles in polymer matrices. Soft Matter.

[89] Sharifzadeh E., Karami M., Ader F. (2023). Formation of nanoparticle aggregates and agglomerates in polymer nanocomposites and their distinct impacts on the mechanical properties. Polym. Eng. Sci..

[90] Zare Y. (2016). Study of nanoparticles aggregation/agglomeration in polymer particulate nanocomposites by mechanical properties. Compos. Part A: Appl. Sci. Manuf..

[91] Deshpande P.A., Delgado A.H.S., Young A.M. (2021). Methacrylate peak determination and selection recommendations using ATR-FTIR to investigate polymerisation of dental methacrylate mixtures. PLoS ONE.

[92] Leggat P.A., Kedjarune U. (2003). Toxicity of methyl methacrylate in dentistry. Int. Dent. J..

[93] Nicholas C.A., Lawrence W.H., Autian J. (1979). Embryotoxicity and fetotoxicity from maternal inhalation of methyl methacrylate monomer in rats. Toxicol. Appl. Pharmacol..

[94] Pemberton M.A., Lohmann B.S. (2014). Risk Assessment of residual monomer migrating from acrylic polymers and causing Allergic Contact Dermatitis during normal handling and use. Regul. Toxicol. Pharmacol..

[95] Pituru S.M., Greabu M., Totan A., Imre M., Pantea M., Spinu T., Tancu A.M.C., Popoviciu N.O., Stanescu I.-I., Ionescu E. (2020). A Review on the Biocompatibility of PMMA-Based Dental Materials for Interim Prosthetic Restorations with a Glimpse into Their Modern Manufacturing Techniques. Materials.

[96] Ahamad Said M.N., Hasbullah N.A., Rosdi M.R.H., Musa M.S., Rusli A., Ariffin A., Shafiq M.D. (2022). Polymerization and Applications of Poly(methyl methacrylate)–Graphene Oxide Nanocomposites: A Review. ACS Omega.

[97] Glazkova E., Bakina O., Rodkevich N., Mosunov A., Evstigneev M., Evstigneev V., Klimenko V., Lerner M. (2022). Antibacterial Properties of PMMA Functionalized with CuFe2O4/Cu2O/CuO Nanoparticles. Coatings.

[98] Siddiqui M.N., Redhwi H.H., Vakalopoulou E., Tsagkalias I., Ioannidou M.D., Achilias D.S. (2015). Synthesis, characterization and reaction kinetics of PMMA/silver nanocomposites prepared via in situ radical polymerization. Eur. Polym. J..

[99] Jabeen S., Gul S., Kausar A., Muhammad B., Farooq M. (2018). An Innovative Approach to the Synthesis of PMMA/PEG/Nanobifiller Filled Nanocomposites with Enhanced Mechanical and Thermal Properties. Polym.-Plast. Technol. Mater..

[100] Guryanova S.V. (2022). Regulation of Immune Homeostasis via Muramyl Peptides-Low Molecular Weight Bioregulators of Bacterial Origin. Microorganisms.

[101] Guryanova S.V., Gigani O.B., Gudima G.O., Kataeva A.M., Kolesnikova N.V. (2022). Dual Effect of Low-Molecular-Weight Bioregulators of Bacterial Origin in Experimental Model of Asthma. Life.

[102] Arjmand B., Alavi-Moghadam S., Sarvari M., Rezaei-Tavirani M., Rezazadeh- Mafi A., Arjmand R., Nikandish M., Nasli-Esfahani E., Larijani B. (2023). Critical roles of cytokine storm and bacterial infection in patients with COVID-19: Therapeutic potential of mesenchymal stem cells. Inflammopharmacology.

[103] Eloseily E.M., Cron R.Q. (2024). Bacteria-Associated Cytokine Storm Syndrome. Cytokine Storm Syndrome.

[104] Nie J., Zhou L., Tian W., Liu X., Yang L., Yang X., Zhang Y., Wei S., Wang D.W., Wei J. (2025). Deep insight into cytokine storm: From pathogenesis to treatment. Signal Transduct. Target. Ther..

[105] Alfei S., Schito G.C., Schito A.M., Zuccari G. (2024). Reactive Oxygen Species (ROS)-Mediated Antibacterial Oxidative Therapies: Available Methods to Generate ROS and a Novel Option Proposal. Int. J. Mol. Sci..

[106] Elbehiry A., Abalkhail A. (2025). Antimicrobial Nanoparticles Against Superbugs: Mechanistic Insights, Biomedical Applications, and Translational Frontiers. Pharmaceuticals.

[107] Gao F., Shao T., Yu Y., Xiong Y., Yang L. (2021). Surface-bound reactive oxygen species generating nanozymes for selective antibacterial action. Nat. Commun..

[108] Wang X., Wang H., Cheng J., Li H., Wu X., Zhang D., Shi X., Zhang J., Han N., Chen Y. (2023). Initiative ROS generation of Cu-doped ZIF-8 for excellent antibacterial performance. Chem. Eng. J..

[109] Zheng G., Tang Z., Peng F. (2025). Ultrasound-activated inorganic nanomaterials to generate ROS for antibacterial applications. Biomater. Sci..

[110] Gudkov S.V., Lyakhov G.A., Pustovoy V.I., Shcherbakov I.A. (2019). Influence of Mechanical Effects on the Hydrogen Peroxide Concentration in Aqueous Solutions. Phys. Wave Phenom..

[111] Temnov A.A., Sklifas A.N., Zhalimov V.K., Sharapov M.G., Fadeev R.S., Kobyakova M.I., Kukushkin N.I., Rogov K.A. (2023). Paracrine Effects of Stem Cell Conditioned Medium on Reactive Oxygen Species Production in Blood Neutrophils in Acetaminophen-Induced Liver Failure. Biophysics.

[112] Goncharov R.G., Filkov G.I., Trofimenko A.V., Boyarintsev V.V., Novoselov V.I., Sharapov M.G. (2020). The Protective Effect of a Chimeric PSH Antioxidant Enzyme in Renal Ischemia–Reperfusion Injury. Biophysics.

[113] Wang L., Li B., Dionysiou D.D., Chen B., Yang J., Li J. (2022). Overlooked Formation of H2O2 during the Hydroxyl Radical-Scavenging Process When Using Alcohols as Scavengers. Environ. Sci. Technol..

[114] Gudkov S.V., Gudkova O.Y., Chernikov A.V., Bruskov V.I. (2009). Protection of mice against X-ray injuries by the post-irradiation administration of guanosine and inosine. Int. J. Radiat. Biol..

[115] Sharapov M.G., Gudkov S.V., Lankin V.Z. (2021). Hydroperoxide-Reducing Enzymes in the Regulation of Free-Radical Processes. Biochemistry.

[116] Kornienko J.S., Smirnova I.S., Pugovkina N.A., Ivanova J.S., Shilina M.A., Grinchuk T.M., Shatrova A.N., Aksenov N.D., Zenin V.V., Nikolsky N.N. (2019). High doses of synthetic antioxidants induce premature senescence in cultivated mesenchymal stem cells. Sci. Rep..

[117] Vostrikova S.M., Grinev A.B., Gogvadze V.G. (2020). Reactive Oxygen Species and Antioxidants in Carcinogenesis and Tumor Therapy. Biochemistry.

[118] Zhevak T., Shelekhova T., Chesnokova N., Tsareva O., Chanturidze A., Litvitsky P., Andriutsa N., Samburova N., Budnik I. (2020). The relationship between oxidative stress and cytogenetic abnormalities in B-cell chronic lymphocytic leukemia. Exp. Mol. Pathol..

[119] Goncharov R.G., Sharapov M.G. (2023). Ischemia–Reperfusion Injury: Molecular Mechanisms of Pathogenesis and Methods of Their Correction. Mol. Biol..

[120] Sies H. (2021). Oxidative eustress: On constant alert for redox homeostasis. Redox Biol..

[121] Ravanat J.L. (2002). Cellular background level of 8-oxo-7,8-dihydro-2′-deoxyguanosine: An isotope based method to evaluate artefactual oxidation of DNA during its extraction and subsequent work-up. Carcinogenesis.

[122] Chiorcea-Paquim A.-M. (2022). 8-oxoguanine and 8-oxodeoxyguanosine Biomarkers of Oxidative DNA Damage: A Review on HPLC–ECD Determination. Molecules.

[123] Matsui A., Ikeda T., Enomoto K., Hosoda K., Nakashima H., Omae K., Watanabe M., Hibi T., Kitajima M. (2000). Increased formation of oxidative DNA damage, 8-hydroxy-2′-deoxyguanosine, in human breast cancer tissue and its relationship to GSTP1 and COMT genotypes. Cancer Lett..

[124] Katerji M., Filippova M., Duerksen-Hughes P. (2019). Approaches and Methods to Measure Oxidative Stress in Clinical Samples: Research Applications in the Cancer Field. Oxidative Med. Cell. Longev..

[125] Li D., Zhang W., Zhu J., Chang P., Sahin A., Singletary E., Bondy M., Hazra T., Mitra S., Lau S.S. (2001). Oxidative DNA damage and 8-hydroxy-2-deoxyguanosine DNA glycosylase/apurinic lyase in human breast cancer. Mol. Carcinog..

[126] De Rosa M., Johnson S.A., Opresko P.L. (2021). Roles for the 8-Oxoguanine DNA Repair System in Protecting Telomeres From Oxidative Stress. Front. Cell Dev. Biol..

[127] Li C., Xue Y., Ba X., Wang R. (2022). The Role of 8-oxoG Repair Systems in Tumorigenesis and Cancer Therapy. Cells.

[128] Kondo S., Toyokuni S., Iwasa Y., Tanaka T., Onodera H., Hiai H., Imamura M. (1999). Persistent oxidative stress in human colorectal carcinoma, but not in adenoma. Free. Radic. Biol. Med..

[129] Kruchinin A.A., Kamzeeva P.N., Zharkov D.O., Aralov A.V., Makarova A.V. (2024). 8-Oxoadenine: A «New» Player of the Oxidative Stress in Mammals?. Int. J. Mol. Sci..

[130] Caraceni P., De Maria N., Ryu H.S., Colantoni A., Roberts L., Maidt M.L., Pye Q., Bernardi M., Van Thiel D.H., Floyd R.A. (1997). Proteins but not Nucleic Acids are Molecular Targets for the Free Radical Attack During Reoxygenation of Rat Hepatocytes. Free. Radic. Biol. Med..

[131] Davies M. (2014). Long-lived reactive species formed on proteins induce changes in protein and lipid turnover. Free. Radic. Biol. Med..

[132] Simpson J.A., Narita S., Gieseg S., Gebicki S., Gebicki J.M., Dean R.T. (1992). Long-lived reactive species on free-radical-damaged proteins. Biochem. J..

[133] Burmistrov D.E., Yanykin D.V., Paskhin M.O., Nagaev E.V., Efimov A.D., Kaziev A.V., Ageychenkov D.G. (2021). Additive Production of a Material Based on an Acrylic Polymer with a Nanoscale Layer of Zno Nanorods Deposited Using a Direct Current Magnetron Discharge: Morphology, Photoconversion Properties, and Biosafety. Materials.

[134] Gudkov S.V., Garmash S.A., Shtarkman I.N., Chernikov A.V., Karp O.E., Bruskov V.I. (2010). Long-lived protein radicals induced by X-ray irradiation are the source of reactive oxygen species in aqueous medium. Dokl Biochem. Biophys.

[135] Bruskov V.I., Karp O.E., Garmash S.A., Shtarkman I.N., Chernikov A.V., Gudkov S.V. (2012). Prolongation of oxidative stress by long-lived reactive protein species induced by X-ray radiation and their genotoxic action. Free. Radic. Res..

[136] Liu X., Novak B., Namendorf C., Steigenberger B., Zhang Y., Turck C.W. (2024). Long-lived proteins and DNA as candidate predictive biomarkers for tissue associated diseases. iScience.

[137] Jena A.B., Samal R.R., Bhol N.K., Duttaroy A.K. (2023). Cellular Red-Ox system in health and disease: The latest update. Biomed. Pharmacother..

[138] Kurutas E.B. (2016). The importance of antioxidants which play the role in cellular response against oxidative/nitrosative stress: Current state. Nutr. J..

[139] Lu X., Mestres G., Singh V., Effati P., Poon J.-F., Engman L., Ott M. (2017). Selenium- and Tellurium-Based Antioxidants for Modulating Inflammation and Effects on Osteoblastic Activity. Antioxidants.

